# Cultural and behavioural drivers of zoonotic disease risk along the wild meat value chain in rural Cameroon

**DOI:** 10.1371/journal.pone.0355275

**Published:** 2026-08-05

**Authors:** Julia E. Fa, Caleb Tata, Lauren Coad, Sagan Friant, Cédric Thibaut Kamogne Tagne, Joseph Mbane, Robert Okale, François Fouda, Pedro Mayor, Stephan M. Funk, Guillermo Ros Brull, Amy Ickowitz

**Affiliations:** 1 Center for International Forestry Research – World Agroforestry (CIFOR-ICRAF), Jalan CIFOR, Bogor, Indonesia; 2 Department of Natural Sciences, Manchester Metropolitan University, Manchester, United Kingdom; 3 Natural Sciences and Environment Hub, University of Gibraltar, Campus Europa Point, Gibraltar; 4 Forests, Resources and People (FOREP), Limbe Botanic Garden, Limbe, South-West Region, Cameroon; 5 Interdisciplinary Centre for Conservation Science, University of Oxford, Oxford, United Kingdom; 6 The Huck Institutes of the Life Sciences, The Pennsylvania State University, University Park, Pennsylvania, United States of America; 7 Center for Infectious Disease Dynamics, The Huck Institutes of the Life Sciences, The Pennsylvania State University, University Park, Pennsylvania United States of America; 8 Fondation Camerounaise de la Terre Vivante (FCTV), Yaoundé, Cameroon; 9 Facultad de Veterinaria, Universitat Autònoma de Barcelona (UAB), Bellaterra, Barcelona, Spain; 10 Nature Heritage, Jersey, Channel Islands; CEA, FRANCE

## Abstract

Wild meat remains a critical source of food security and income in tropical regions, yet it poses significant public health risks due to the potential for zoonotic disease transmission. To better understand these risks, we conducted 2,374 structured interviews with hunters and food preparers across 44 rural villages in Cameroon and surveyed 64 wild meat vendors in four regional markets. This study explores prevailing wild meat handling practices and perceptions of disease risk, providing essential insights for designing targeted interventions to safeguard human health and livelihoods. Hunting patterns varied by ethnicity: a higher percentage of Indigenous Baka reported hunting than Bantu, and Baka hunters were more likely to target bats, a high-risk taxonomic group. Gender, age, and ethnicity also shape behaviours. Women and older individuals were generally less likely to hunt or handle animals exhibiting clinical abnormalities. However, notable differences were observed between ethnic groups. Among the Indigenous Baka, women frequently participated in hunting, particularly by setting snares, whereas Bantu women seldom did so. Across both groups, women were more likely than men to take home carcasses found dead and to hunt small mammals such as rodents. These distinctions highlight how gendered and cultural norms influence patterns of wildlife contact and exposure to zoonotic risk. Hygiene standards during the handling and processing of wild meat were generally very low. Fewer than 1% of household respondents reported using any protective equipment, and only 6% cleaned butchering surfaces with soap. A substantial number admitted to consuming or selling meat from animals that appeared visibly diseased, exhibiting signs such as abnormal organs, discolouration, or unusual odours. Injuries sustained during butchering were also commonly reported, further compounding health risks. Concern about disease was relatively low, reported by only 14.7% of households. However, it was associated with a modest increase in the likelihood of handwashing (by 5 percentage points) and a more substantial increase in the possibility of avoiding contact with dead animals (by 16 percentage points). Ethnic and gender differences influenced both concerns about disease risk and behaviours. These findings highlight the importance of designing public health strategies grounded in local sociocultural realities, aimed at reducing zoonotic risk while safeguarding livelihoods and respecting the economic and cultural roles that wild meat plays within communities.

## Introduction

Wild meat plays a vital role in the livelihoods, food security, and cultural practices of many rural communities across tropical and subtropical regions worldwide [1–3]. In Central Africa, wild animals provide an important source of protein and income, particularly in remote forest regions where alternative livelihood opportunities and domestic livestock production are limited. At the same time, the harvesting, handling, and consumption of wild animals create opportunities for zoonotic pathogen transmission from wildlife to humans [4].

Research on zoonotic disease risks in wildlife systems has often focused on ecological drivers such as biodiversity loss and land-use change. However, cultural practices, livelihood strategies, and behavioural norms strongly influence how people interact with wildlife and therefore shape patterns of pathogen exposure. Understanding these social and behavioural dimensions is essential for designing disease prevention strategies that are both culturally appropriate and locally feasible.

Several zoonotic diseases of wildlife origin have been documented in Cameroon, underscoring the country’s vulnerability to pathogen emergence. Fruit bats are natural reservoirs for filoviruses such as Ebola and Marburg [5,6], while non-human primates carry simian retroviruses closely related to HIV and other pathogens capable of cross-species transmission [7,8]. Small mammals, including rodents and civets, are also implicated in bacterial infections such as leptospirosis, salmonellosis, and plague [9–11]. Human exposure to these pathogens frequently occurs during hunting, butchering, transport, and food preparation, when contact with animal blood, bodily fluids, or contaminated surfaces provides opportunities for transmission. These repeated and close interactions along wild meat value chains make rural Cameroon an important setting for investigating zoonotic disease dynamics and the human behaviours that shape them.

Zoonotic spillover events have repeatedly demonstrated their capacity to escalate into major global health and economic crises. The 2003 SARS outbreak, the West and Central African Ebola epidemics, and the COVID-19 pandemic illustrate how emerging pathogens can spread rapidly across continents, causing widespread mortality and severe economic disruption [12–15]. In response, some policymakers have proposed banning or severely restricting wildlife trade to prevent future pandemics [16–18]. However, conservationists, social scientists, and public health experts have warned that indiscriminate bans could exacerbate poverty, undermine food sovereignty, and alienate communities whose cooperation is essential for disease prevention [19–23].

A more balanced approach requires a deeper understanding of the social, cultural, and behavioural dimensions of zoonotic risk. Disease transmission is not only a biological process but also a social one, shaped by people’s perceptions, beliefs, and everyday practices. Studies across Africa show that many individuals are unaware of the zoonotic potential of wild meat or perceive little risk in its consumption [24–26]. In Nigeria and Ghana, wild animals are sometimes viewed as “purer” than domestic livestock and therefore safer to eat [27,28], while in the Democratic Republic of the Congo vendors and consumers often distinguish between “clean” and “unclean” animals according to cultural norms rather than biomedical criteria [29]. In Cameroon, symptoms such as diarrhoea, fever, or stomach pain are frequently normalised or attributed to spiritual or environmental causes rather than foodborne infection [30]. These interpretations influence how people evaluate health threats and whether they adopt preventive practices. When wild meat is essential for nutrition or income, individuals may rationally downplay potential disease risks in favour of immediate livelihood needs.

Understanding how local actors perceive and respond to zoonotic risk is therefore critical for designing effective and context-sensitive disease prevention strategies. Despite growing recognition of the importance of behavioural drivers of spillover risk, relatively few studies have examined how everyday practices along wild meat value chains influence the transmission of potential pathogens in Central Africa. In this study, conducted in rural Cameroon, we investigate how individuals involved in the wild meat value chain—including hunters, vendors, and food preparers—perceive disease transmission risks and how these perceptions influence behaviours related to wildlife handling and consumption. We also examine how risk perceptions and practices vary across gender, ethnicity (Bantu and Indigenous Baka), and age groups. By analysing these cultural and behavioural dimensions, this study aims to provide evidence to inform risk communication strategies and community-based interventions designed to reduce zoonotic disease risks, while recognising the socio-economic importance of wild meat systems.

## Methods

### Study area

**Fig 1:**
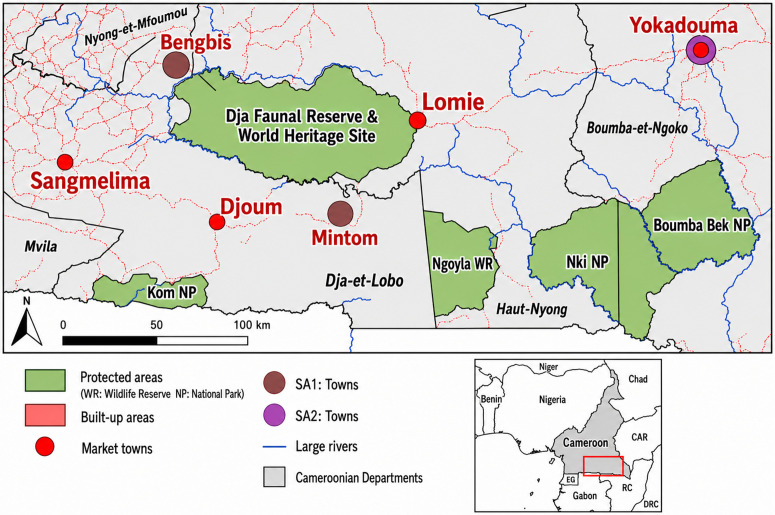
Study area. The sites of the first study area, SA1, are villages around the towns of Mintom (South Dja), Bengbis (West Dja) and Lomié (East Dja). The second study area, SA2, comprises villages between Boumba Bek National Park and Yokadouma. The regional markets Lomié, Djoum, Sangmelima, and Yokadouma were also surveyed. The small, bottom map shows the spatial context of Cameroon and the study area, which is shown in detail in the top map (RC: Republic of Congo, DRC: Democratic Republic of Congo, EG: Equatorial Guinea). The figure was assembled from openly licensed vector datasets. Populated-place and protected-area information derived from OpenStreetMap data is available under the Open Database License (ODbL 1.0), with attribution to © OpenStreetMap contributors. Country and administrative boundaries were obtained from the World Bank Official Boundaries dataset, which is licensed under CC BY 4.0. The locations classified as market, SA1 and SA2 towns were generated from the authors’ study data. Full source and licence information for each external spatial dataset has now been included in the Fig 1 caption.

Our research was conducted in two locations—Study Area 1 (SA1) and Study Area 2 (SA2)—SA1 is situated within the South Region of Cameroon, while SA2 is in the East Region (Fig 1).

**Study Area 1 (SA1)** includes 33 villages around the Dja Biosphere Reserve (DBR), a UNESCO World Heritage Site established in 1950. Spanning approximately 5,260 km^2^, the DBR is one of Central Africa’s largest and best-preserved tropical rainforest reserves (UNESCO, 2021). It is bordered on three sides by the Dja River, which acts as a natural boundary and helps preserve the area’s ecological integrity. The region is home to a mixture of Baka Pygmies and various Bantu ethnic groups, including the Boulou, Fang, Zaman, Badjoué, and Nzimé, many of whom rely heavily on hunting for subsistence and income generation [31].

While subsistence hunting is permitted within the reserve’s buffer zones, commercial hunting and agricultural expansion are strictly prohibited [32]. Human population density in SA1 was estimated at 1.5 people per km^2^ in 2001 [33] but has increased in recent years due to natural population growth and infrastructure development, such as dam construction and agroforestry plantations.

SA1 was further divided into three sampling clusters to capture geographical and socio-cultural variation: **South Dja** (11 villages located south of the reserve near Mintom), **West Dja** (12 villages situated west of the reserve, close to Bengbis) and **East Dja** (11 villages found east of the reserve, near Lomié).

**Study Area 2 (SA2)** comprises 10 villages near Boumba Bek National Park in Cameroon’s East Region. This area lies between the Boumba River and the town of Yokadouma. Officially designated in 2005, Boumba Bek National Park covers approximately 2,382 km^2^ and is recognised for its high biodiversity, including significant populations of forest elephants and other threatened species [34].

The human population in SA2 consists primarily of Baka Pygmies and the Konabembe, a Bantu-speaking community. The Konabembe are traditionally agriculturalists, while the Baka—historically nomadic hunter-gatherers—have undergone a gradual socio-economic transition [35]. Many Baka now farm or work as agricultural labourers in Bantu households, often in exchange for food, goods, or limited monetary compensation.

Both regions are ecologically diverse, dominated by dense tropical rainforests that form part of the Congo Basin, one of the world’s most extensive and biologically rich forest systems [36,37]. These forests provide critical habitat for a wide array of plant and animal species, some of which are globally threatened or endemic.

SA1 and SA2 represent complementary ecological and socio-cultural contexts, allowing us to assess patterns across varied settings. This enhances the generalizability of our findings and reduces the risk of location-specific bias.

### Research design

We carried out a structured quantitative study to examine wild meat practices and perceptions of disease risk. Data collection concentrated on three key groups: rural hunters, household food preparers, and wild meat vendors operating in the markets of Lomié, Djoum, Sangmelima, and Yokadouma (Fig 1).

### Pre-study engagement

Before data collection, we conducted a scoping visit to both study areas to initiate dialogue with key stakeholders and build trust within the communities. Consultations were held with administrative authorities at the regional, divisional, and sub-divisional levels, including Governors, Divisional Officers, and Sub-Divisional Officers, as well as with forestry and wildlife officials, conservation organisations, local councils, and village chiefs. These meetings introduced the study, clarified its objectives, and helped secure community support for the research.

Research permits were obtained from the Ministry of Scientific Research and Innovation (MINRESI), and authorization to conduct research in protected areas was obtained from the Ministry of Forestry and Wildlife (MINFOF) prior to fieldwork. These permits ensured that all research activities complied with national regulations and ethical standards governing scientific research in Cameroon.

Stakeholder feedback also informed the identification of villages with notable hunting activity in the vicinity of the Dja Biosphere Reserve and Boumba Bek National Park. In addition, our longstanding and intensive collaboration with Baka and Bantu hunters in the Djoum area has played a key role in fostering mutual trust and facilitating community engagement with local communities and local and national administrative authorities [20,36,37–39].

### Village selection

Villages were stratified into three demographic categories based on their ethnic composition: predominantly Bantu, mainly Baka, and mixed-population settlements. This stratification enabled the study to compare practices and perceptions across cultural groups and generate insights that are more broadly representative of the region. Eligible villages had at least 100 households. Within each village, households were randomly selected to participate in structured surveys.

### Data collection methods

We collected data on hunting practices, wild meat handling behaviours, and perceptions of disease risk using structured questionnaires administered to selected households and wild meat vendors (see Supplementary Materials). Interviews with hunters also gathered information on the primary methods used to capture wildlife, including snaring, firearm hunting, opportunistic capture, and trapping techniques. Although hunting methods were not the focus of the present analysis, they were documented because they influence the nature and frequency of human–wildlife contact and therefore may affect hunters’ potential exposure to zoonotic pathogens.

The study focused on key actors involved in the wild meat value chain. For the purposes of this study, **hunters** were defined as household members actively engaged in hunting activities, whether occasionally or professionally. **Food preparers**, typically women, were primarily responsible for preparing meals in the household and, therefore, frequently handled raw wild meat. **Vendors** were individuals selling wild meat in local markets, usually as part of informal or small-scale commercial networks. In addition, wildlife products are sometimes moved through **intermediaries**, defined as individuals who purchase wild meat from hunters in villages and transport or redistribute it to urban or peri-urban markets, thereby linking hunters to market vendors.

### Sample sizes

Using population data from the 2005 census (BUCREP, 2005) and consultations with local authorities, we estimated an average of 1,100 households in SA1 (Lomie, Bengbis, and Mintom). In SA2, the population of the selected villages was estimated at 1,549 households according to the 2012 census. For sample size estimation, we applied the following formula [40]:


$$\begin{array}{c} s=\;\frac{X^{\text{2}}\times N\times P\times (\text{1}-P)}{d^{\text{2}}\times (N-\text{1})+X^{\text{2}}\times P\times (\text{1}-P)} \end{array}$$ (1)

where:

- ***X* = 1.96** (Z-value for 95% confidence)
- ***N* = 1100** (population size)
- ***P* = 0.5** (assumed distribution of variables of interest)
- ***d* = 0.05** (margin of error)

The minimum required sample size was 285 households per site in SA1 and 308 households per site in SA2.

### Data collection and analysis

**Participant selection and survey administration.** We interviewed a total of 2,374 individuals, classified as either hunters or household food preparers. To complement these data, we also conducted structured interviews with all wild meat vendors operating in the four principal regional markets—Lomié, Djoum, Sangmelima, and Yokadouma—ensuring comprehensive representation of key actors along the wild meat value chain. In total, 64 vendors were surveyed.

**Data collection procedures.** The household and vendor survey questionnaires (S1 and S2 Tables) were specifically developed for this study and administered between 14 October and 4 November 2022. Data were collected using KoboCollect (www.kobotoolbox.org), a mobile platform compatible with tablets and smartphones that enables real-time data entry and synchronisation. A team of five Cameroonian enumerators, fluent in French and the local languages relevant to each study site, conducted the interviews at each study site. Before data collection, enumerators received training from the project team and participated in a pretest phase that informed revisions to enhance the clarity and reliability of the questionnaires.

### Statistical analysis

Following data collection, survey responses were exported from the Kobo Collect platform and transferred to Microsoft Excel for data cleaning and organisation. The processed dataset was then imported into **Stata 17** [41] and **R** [42] for comprehensive statistical analysis, including descriptive (Table 2) and inferential methods.

We began with descriptive analyses to explore key demographic and behavioural patterns across respondent groups. We then used regression models to examine how individual characteristics, such as sex and ethnicity, independently influenced the likelihood of hunting. Dummy variables representing each study site were included to control for location-specific effects not captured by other covariates.

**Hunting model.** We used a logistic regression model to examine predictors of hunting behaviour. The dependent variable was whether the respondent engaged in hunting, and the explanatory variables included demographic characteristics (e.g., age group, sex, ethnicity) and site-specific fixed effects. This allowed us to assess which individual attributes were significantly associated with the likelihood of hunting, controlling for confounding variables. We used the following regression formula:


$$\begin{array}{c} ogit\;Pr(hunt=\text{1})=\propto +\beta_{\text{1}}Baka+\beta_{\text{2}}Female+\;\beta_{\text{3}}older+\;\beta_{\text{4}}young+\;\beta_{\text{5}}Bengbis+\;\beta_{\text{6}}Mintom+\;\beta_{\text{7}}Yokadouma+\varepsilon \end{array}$$

**High-risk taxa models.** The structured questionnaire included items probing the frequency with which respondents hunted taxa recognised as high-risk for zoonotic disease transmission, specifically rodents, primates, and bats. Participants selected from five response options: *Never, Rarely, Sometimes, Often,* and *Always*. Because the distinction between ‘rarely,’ ‘sometimes,’ and ‘often’ is highly subjective, we reclassified the responses into binary categories of ‘ever’ and ‘never’ for the regression analysis. We ran three logistic regression models—one for each taxonomic group—using the same independent variables as in the main hunting model. These models allowed us to assess how different respondent characteristics were associated with hunting high-risk taxa.

**Marginal effects.** We calculated and reported **marginal effects** for all logistic and multinomial logistic regressions to enhance interpretability. Marginal effects represent the change in the predicted probability of the outcome variable associated with a one-unit change in a predictor variable, holding all other variables constant. These were expressed as predicted probabilities, facilitating more explicit comparisons across subgroups.

**Fig 2:**
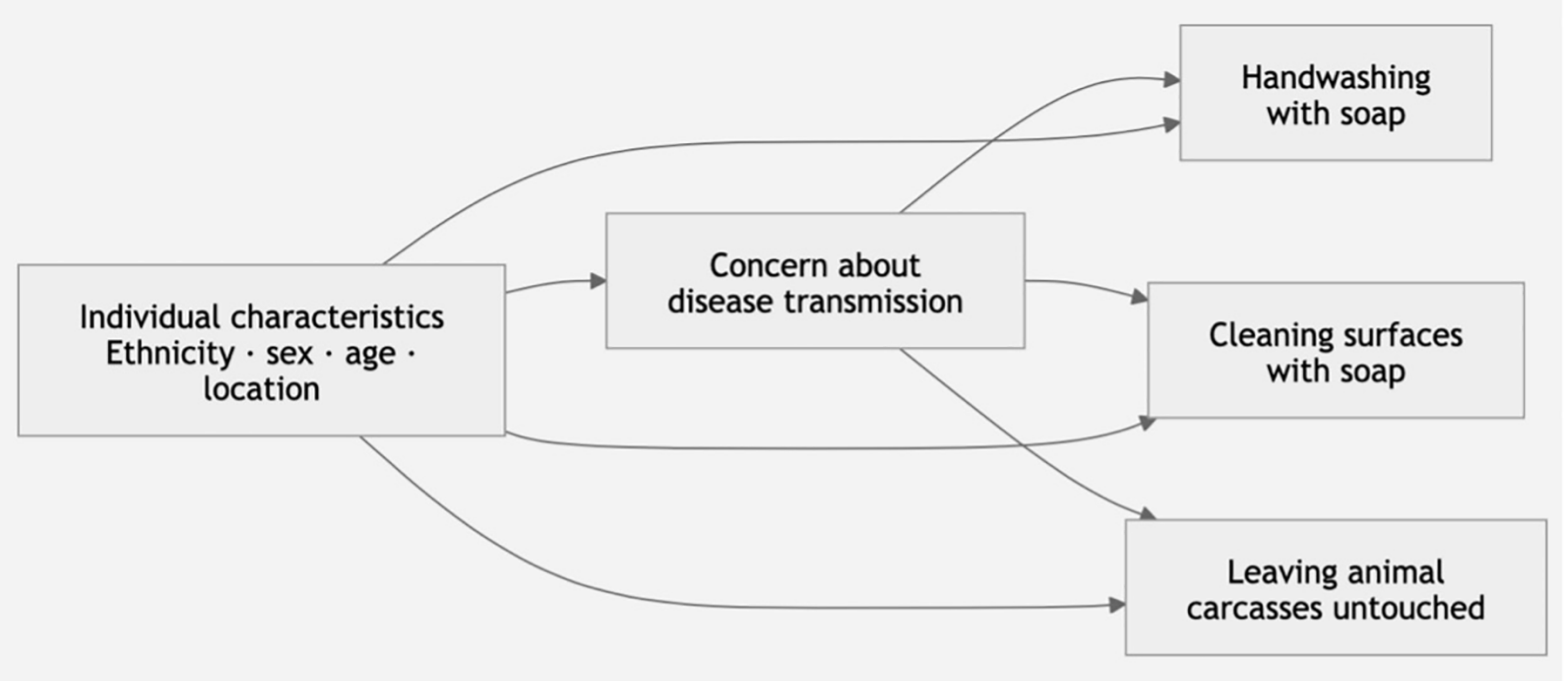
Conceptual structure of the structural equation model used to estimate direct and indirect relationships among individual characteristics, concern about disease transmission and protective behaviours. Individual characteristics were modelled as predictors of concern and protective behaviours. Concern was specified as a potential mediator. Thus, characteristics could influence behaviour directly (c“‘”) or indirectly through concern (a × b). Handwashing, cleaning surfaces with soap and leaving animal carcasses untouched were modelled as [separate outcomes/indicators of a common protective-behaviour construct].

**GSEM modelling.** We also investigated the relationship between concern about zoonotic risk, specifically related to contact with animal blood, and the adoption of risk-mitigating behaviours. Recognising that demographic factors (gender, ethnicity and age) may influence both risk perception (indirect effects) and actual behaviours (direct effects), we used Generalised Structural Equation Modelling (GSEM) to capture these complex relationships [41]. Structural equation models combine elements of factor analysis with regression modelling to account for direct, indirect, and mediated effects between observed and latent variables. The factor analysis component identifies the latent variables, while the regression component models the relationships between the latent and observed variables to estimate the outcomes. We chose GSEM because it allows for modelling non-linear relationships among variables and accommodates different types of dependent variables, such as binary data, as in our model [43]. The conceptual framework underpinning the GSEM analysis is presented in Fig 2.

Three behavioural outcomes were modelled in the GSEM analysis: (i) washing hands with soap after handling wild meat, (ii) cleaning butchering surfaces with soap, and (iii) avoiding the collection of dead animals found in the forest.

To facilitate interpretation of model results, marginal effects derived from the GSEM regressions are presented as predicted probabilities.

Marginal effects from the GSEM regressions were also presented as probabilities to facilitate interpretation.

### Ethics and inclusivity in global research

Ethical approval for this study was obtained from the National Ethical Committee of Research for Human Health in Cameroon (Approval No: 2022/08/1481/CE/CNERSH/SP). Prior to fieldwork, research permits were obtained from the Ministry of Scientific Research and Innovation (MINRESI), and authorization to conduct research in protected areas was granted by the Ministry of Forestry and Wildlife (MINFOF). These approvals ensured that all research activities complied with national regulations governing scientific research in Cameroon.

Before participation, informed consent was obtained from all respondents. Participants were informed about the objectives of the study, the voluntary nature of their participation, and their right to decline to answer any question or withdraw from the interview at any time without penalty. Interviews were conducted by trained Cameroonian enumerators fluent in French and the relevant local languages to ensure that participants clearly understood the study and the consent process. All responses were anonymised, and confidentiality of participants’ information was strictly maintained.

Participants were not notified in advance about the provision of a small appreciation gift. The questionnaire focused primarily on factual and descriptive topics, including hunting practices, carcass handling, wildlife trade, household meat consumption, hygiene behaviours, and perceptions of animal health. This approach was intended to minimise potential response bias while maintaining transparency regarding the purpose of the study. As a token of appreciation for their time, participants received two bars of soap valued at approximately 700 XAF (USD $1.16) after completing the interview. Although some participants may have become aware of the gift through word of mouth, its modest value and distribution only after the interview were intended to minimise the likelihood that responses would be influenced by expectations of compensation.

Additional information regarding the ethical, cultural, and scientific considerations specific to inclusivity in global research is included in the Supporting Information (S3 Checklist).

## Results

A total of 1,186 hunters, 1,213 food preparers, and 64 wild meat vendors were surveyed across 44 villages in the four study landscapes surrounding the Dja Faunal Reserve and Boumba Bek National Park (Table 1). Respondents were relatively evenly distributed across East Dja, West Dja, South Dja, and Boumba Bek, with each site contributing between 277 and 330 hunters and 292 and 317 food preparers.

**Table 1:** Number of hunters, food preparers, and vendors surveyed in the study in southeastern Cameroon.

Sites	Villages	Hunters	Food preparers	Vendors
East Dja	11	277	300	22
West Dja	12	292	304	16
South Dja	11	287	292	18
Boumba Bek	10	330	317	9
Total	44	1,186	1,213	64

Wild meat vendors were surveyed in local villages and roadside markets operating within the study areas. Because the number of vendors is generally small and variable, all vendors selling wild meat encountered during the survey were approached and interviewed, yielding a near-census of active vendors during the survey period. Consequently, vendor numbers were lower than those for hunters and food preparers, ranging from 9 in Boumba Bek to 22 in East Dja.

The vendor counts reported in Table 1 represent the number of individual vendors interviewed, not the number of markets surveyed. For example, in West Dja, surveys were conducted across 17 markets, where 16 vendors were interviewed; one market had no active wild meat vendors at the time of the survey.

### Descriptive statistics: Survey respondent characteristics

Our household survey captured a nearly equal gender distribution among respondents, with 50.2% identifying as male and 49.8% as female (Table 2). Most respondents (76.9%) identified as Bantu, whereas 23.1% identified as Baka. The predominant occupation was cropping (80.4%). Even though only 5.8% reported hunting as their main livelihood activity, 47.6% reported sometimes hunting, with most male respondents (83.8%) reporting sometimes hunting.

**Table 2:** Demographic variables and hunting behaviour (percentages with standard errors).

Variables	I. Household	II. Hunter	III. Vendors
	Survey	Sub-sample	
**Gender**			
Male	50.2 (1.0)	88.5 (0.95)	3.1 (2.2)
Female	49.8 (1.0)	11.5 (0.95)	96.9 (2.2)
**Ethnicity**			
Baka	23.1 (0.9)	30.7 (1.4)	
Bantu	76.9 (0.9)	69.3 (1.4)	
**Age Groups (years)**			
18–25	15.1(0.7)	14.9 (1.1)	
26–40	39.4 (1.0)	39.9 (1.5)	
41–60	35.4 (1.0)	39.1 (1.5)	
Above 60	10.1(0.6)	6.1 (0.7)	
**Main Occupation**			
Crop farming	80.4(0.8)	79.4 (1.2)	
Hunting	5.8 (0.5)	12.1(1.0)	
Fishing	0.5 (0.15)	0.4 (0.2)	
Home maker	3.2 (0.4)	0.4 (0.2)	
Other	10.1(0.6)	7.7 (0.8)	
**Wild Meat Handling Practices**			
Hunt	47.6 (1.0)		
Butcher wild meat	86.4 (0.7)		
Use protective clothing/gloves	0.63 (0.2)		25.0 (5.4)
Concerned about risks	14.7 (35.4)		26.6 (5.5)
Wash hands with soap after handling	55.4 (0.01)		71.9 (5.6)
Wash surfaces with soap/alcohol	6.2 (0.50)		37.5 (6.1)
Leave dead animals	20.7 (40.5)		
**Injuries**			
Injured while butchering/preparing	50.8 (1.5)		92.2 (7.8)
**Handling Diseased Meat**			
Eat/sell meat with lesions/signs of illness	45.4 (1.0)		97.8 (2.2)
**Ever Hunt or Sell High Risk Taxa**			
Bats		20.8 (1.2)	
Primates		60.1(1.5)	
Rodents		98.3 (0.4)	
**Number of Observations**	**2,373**	**1,131**	**64**

### Wild meat handling practices and exposure to risk

Wild meat butchering was a daily task for 86% of all respondents. Vendors consistently received meat from hunters or intermediaries, either fresh or smoked. Most vendors—84%—butchered animals directly in the market, routinely handling carcasses and being exposed to blood and other bodily fluids (Table 2). Despite the evident risks, protective clothing was virtually absent: only 0.92% of respondents consistently used protective equipment, whereas 25% reported occasional use.

Hygiene practices were uneven. Although 55% of household respondents and 85% of vendors reported washing their hands with soap after handling wild meat, only a small proportion cleaned butchering surfaces with soap or disinfectants—6.2% among households and 12.5% among vendors (Table 2). Cutting surfaces were often rudimentary: wooden planks, leaves, or sheets of cardboard, the latter two typically discarded only at the end of the day.

Injuries during meat preparation were common. Among household respondents, 56.6% had suffered injuries, usually from knives, machetes, or animal bites and scratches. Vendors reported even higher injury rates: 96% had at least one accident during butchering at some point in their lives. Of those injured, 35% used basic first aid like bandages, 10% used no treatment at all, 8% resorted to traditional remedies such as applying salt, saliva, or pepper, and 46% sought formal medical care, whether from a hospital, pharmacy, or using antiseptics like alcohol or betadine (Table 2).

Risky consumption practices were widespread. Nearly half of household respondents—45.4%—admitted to consuming meat from animals that showed visible signs of illness. Vendors reported an even higher risk tolerance, with 97.8% stating they sold meat from animals exhibiting signs of infection, often removing worms by hand and then selling the rest of the meat (Table 2).

A significant number of respondents reported handling animals with clinical abnormalities, including worms, maggots, leeches, ticks, abscesses, wounds, or lesions. In some households, visibly infected organs were fed to domestic dogs while the remaining meat was cooked and consumed (Table 2). During hunting trips, 72% of hunters reported encountering dead animals at least once. Of these, 55% left the carcasses undisturbed, whereas 45% reported retrieving, eating, or selling them (Table 2). Evisceration, particularly of rodents and bats, frequently occurred at home, posing potential health risks to family members. Organ inspection practices were poor: nearly half (48.6%) of respondents never inspected internal organs for abnormalities, and among those who did, 36.8% reported removing only the visibly affected parts before cooking the rest.

### Hunting and trade of high-risk taxa

Taxa known to be particularly associated with elevated zoonotic risk were frequently hunted. Rodents were the most hunted group, with 98% of hunters reporting having hunted them. Primate hunting was reported by 60% of respondents, while 21% reported hunting bats, based on cumulative responses across all frequency categories (Table 2). Among vendors, trade in high-risk taxa was widespread. Nearly all vendors sold primates (96.9%) and rodents (90.5%), while 8% reported selling bats.

### Risk-mitigation behaviours

Protective behaviours to reduce zoonotic risk were limited. Fewer than 1% of hunters and food preparers reported using protective gear such as gloves or aprons when handling dead animals. Among vendors, 25% used some form of protection, including aprons (50%), designated clothing (25%), and gloves (12%) (Table 2). While handwashing with soap was more commonly reported by 55% of household respondents and 85% of vendors, proper surface cleaning remained rare. Only 6% of hunters and food preparers, and 12.5% of vendors, cleaned butchering surfaces using soap or disinfectants.

Waste management practices were also inadequate. Although 69% of vendors reported designated waste disposal sites, these were often open bush areas near the markets. Butchering surfaces used in the market included disposable materials such as cardboard, which were typically discarded at the end of the trading day (Table 2).

### Statistical modelling results

Regression analysis identified several demographic correlates of hunting behaviour. Baka respondents had a 17 percentage-point higher likelihood of hunting than their Bantu counterparts, a statistically significant difference. Gender played a major role: female respondents were 73 percentage points less likely to hunt than male respondents. Age was also significant, with individuals over 60 being 15 percentage points less likely to hunt than those aged 18–25. No statistically significant differences were found between the remaining age categories (25–40 and 41–60 years). Hunting behaviour varied considerably across study sites, as detailed in Table 3.

**Table 3:** Characteristics of hunters: marginal effects from logistic regression.

Independent variables	Marginal effects (standard errors)
**Baka**	0.173*** (0.023)
**Female**	−0.724*** (0.023)
**‘older’ (>60 yrs)**	−0.147*** (0.029)
**‘young’ (18–25 yrs)**	−0.008 (0.018)
**Bengbis (West Dja)**	−0.049 (0.022)
**Mintom (South Dja)**	0.047 (0.030)
**Yokadouma (SA 2)**	0.071** (0.023)
**Wald chi2(7)** (prob>chi^2^)	268.31 (0.000)
**N**	2,374

When examining hunting of high-risk taxa, Table 4 shows that Baka respondents were 9.8 percentage points more likely to hunt bats than Bantu respondents, though no ethnic differences were observed for hunting rodents or primates. Gender was not associated with differences in the hunting of any of the high-risk groups. Age, however, was associated with primate hunting: respondents aged 60 or older were 12.9 percentage points less likely to hunt primates than their younger counterparts. No other age-related differences were statistically significant.

**Table 4:** Hunting high-risk animals (Bats, Primates, and Rodents): Marginal Effects and Standard Errors from Logistic Regressions.

	I. Ever hunt Bats	II. Ever hunt Primates	III. Ever hunt Rodents
**Baka**	0.098** (0.035)	0.023 (0.053)	0.004 (0.010)
**Female**	0.006 (0.053)	−0.071 (0.082)	−0.009 0.021)
**>60**	−0.059 (0.046)	−0.129* (0.064)	−0.014 (0.038)
**Young**	−0.013 (0.033)	0.011 (0.042)	0.000 (0.015)
**West Dja**	0.295*** (0.065)	−0.099 (0.066)	Not estimable
**South Dja**	−0.051 (0.065)	0.174*** (0.041)	0.017 (0.010)
**Boumba Bek**	0.306*** (0.063)	−0.035 (0.060)	−0.013 (0.014)
Wald chi2(7) (prob>chi2)	80.42 (0.00)	51.58 (0.00)	6.06 (0.42)
**N**	1,131	1,129	918

### Perceptions of disease risk and behavioural associations

Concern about zoonotic disease risk was generally low across all groups. Only 14.7% of household respondents and 26.6% of vendors reported being worried about disease risks from wild meat. Among hunters and food preparers, 85% expressed no concern, rising to 89% among vendors (Table 2).

Results from a GSEM revealed associations between concern and specific behaviours. Expressing concern about disease risk was associated with a 5 percentage-point higher likelihood of leaving dead animals undisturbed and a 16.1 percentage-point higher likelihood of washing hands with soap after handling meat. However, no statistically significant relationship was found between concern and surface cleaning after butchering.

Ethnic and gender differences emerged regarding both concern and risk-mitigating behaviours. Baka respondents were 4.9 percentage points more likely than Bantu respondents to express concern about zoonotic disease and 5.7 percentage points more likely to leave dead animals undisturbed. However, they were also 16.1 percentage points less likely to wash their hands with soap. Gender differences followed a different pattern: women were four percentage points more likely than men to express concern and 9.8 percentage points more likely to wash their hands with soap after handling wild meat. Yet women were 34.6 percentage points less likely than men to leave dead animals undisturbed and were 3.2 percentage points more likely to clean butchering surfaces with soap. Age, in contrast, was not significantly associated with levels of concern about disease risk (Table 5).

**Table 5:** Marginal Effects from GSEM model: Risk perceptions and precautions.

	I. Concerned	II. Leave animals found dead	III. Wash hands with soap	IV. Wash butchering surface with soap
**Concerned**		0.050* (0.022)	0.168*** (0.029)	0.026 (0.014)
**Baka**	0.0457*** (0.018)	0.059** (0.018)	−0.156*** (0.023)	−0.018 (0.026)
**Female**	0.041*** (0.015)	−0.346*** (0.016)	0.097*** 0.019)	0.034*** (0.010)
**>60**	−0.027 (0.029)	−0.082** (0.056)	−0.136*** (0.034)	−0.004 (0.0173)
**Young**	0.030 (0.019)	−0.024 (0.024)	0.028 (0.029)	−0.037** (0.014)
**West Dja**	−0.000 (0.020)	−0.026 (0.062)	−0.004 (0.031)	0.025 (0.015)
**South Dja**	−0.086** (0.023)	0.073*** (0.021)	−0.230*** (0.028)	−0.028 (0.014)
**Boumba Bek**	0.025 (0.020)	−0.022 (0.045)	−0.217*** (0.027)	0.044** (0.047)
**Log likelihood**		−1848	−2343	−1401
**N**	2,261	2,261	2,261	2,261

## Discussion

When individuals engage in risky behaviours, two primary psychological mechanisms may be at work: knowledge deficit or risk habituation. These mechanisms represent distinct pathways that explain why people continue hazardous practices despite objective danger. The knowledge deficit model suggests that risky behaviour stems from a lack of awareness or understanding about potential hazards [44]. The model implies that providing accurate information that improves knowledge will lead to behavioural change. For example, a hunter who handles bat carcasses without protection may be unaware of the potential for viral transmission of pathogens like Ebola or coronaviruses. This individual might readily adopt protective measures once informed and convinced of specific risks. Conversely, risk habituation (or risk normalisation) occurs when individuals become desensitised to hazards through repeated exposure without apparent negative consequences [45]. This phenomenon, sometimes called the “experience trap,” leads people to discount risks based on their history of uneventful exposures. For example, a wild meat vendor who has processed carcasses for decades without knowingly experiencing illness related to this behaviour may develop a sense of invulnerability, disregarding safety protocols even when aware of potential dangers. As noted by Loewenstein and Mather [46], personal experience often outweighs statistical information in risk assessment. Both knowledge deficit and risk habituation mechanisms will be influenced by the population’s perception of health problems due to the regular coexistence with moderate clinical signs, which can lead to the local population to assume, for example, that the occurrence of mild diarrhoea is normal; therefore, no risk is perceived and no preventive or curative measures will be taken to mitigate these clinical signs.

Low concern about zoonotic disease risk—reported by only 15% of household respondents—presents an interpretive challenge. This finding alone does not allow for a clear distinction between a lack of knowledge and risk habituation. However, focus group discussions conducted alongside the survey, though not reported in this paper, strongly indicate widespread knowledge gaps concerning disease transmission. At the same time, a subset of individuals who expressed concern and reported adopting risk-mitigating behaviours suggests that relevant knowledge does exist within these communities. This knowledge has not translated into consistent preventive action across the broader population of households, hunters, butchers, and vendors. These observations point to a complex interplay between knowledge, risk perception, and habituation that warrants deeper investigation.

In our study region, risky behaviours are prevalent throughout the wild meat value chain. Hunters frequently target potentially high-risk taxa: 98% hunt rodents, 60% hunt primates, and 21% hunt bats. The differential targeting of species—higher for rodents than for primates or bats—should not be interpreted solely as an indicator of disease awareness. Rather, this pattern likely reflects a combination of prey preference and practical constraints. Hunting primates often requires specialised equipment, such as firearms, whereas bat hunting involves nets or other targeted methods, whereas rodent hunting primarily relies on cheap, widely available wire traps.

Multiple mechanisms are known to shape human behaviour in the face of health risks, often operating simultaneously [47]. One such factor is optimistic bias, in which individuals systematically underestimate their own vulnerability relative to others, even when they are aware of the risks [48]. Cultural-cognitive influences also play a role, as perceptions of risk are shaped by local worldviews and social norms that may prioritise economic necessity or tradition over safety [49]. In addition, the affect heuristic suggests that emotional responses to hazards—such as fear, familiarity, or trust—can override logical assessments, especially when the risky behaviour provides immediate benefits, like food or income [50,51]. Finally, practical constraints often limit the ability to act on knowledge: even when individuals understand the risks, structural barriers such as limited access to protective equipment or the absence of viable economic alternatives can make safer choices unattainable [52].

As Renn [53] argues, effective risk communication must address both knowledge deficits and experiential factors, while recognising that risk behaviours are embedded within broader social, cultural, and economic contexts that influence decision-making processes.

In wildlife harvesting communities specifically, Kamins et al. [54] found that hunters often recognised disease risks but continued hazardous practices due to economic necessity, social expectations, and a history of exposure without apparent consequences, demonstrating how knowledge and habituation interact within complex social ecosystems.

### Risk behaviours for zoonotic disease transmission

Our findings reveal a suite of behaviours among hunters, food preparers, and vendors that significantly increase the risk of zoonotic disease transmission. These include the types of animals hunted, handling practices, hygiene behaviours, the absence of protective equipment, and concern (or lack of concern) regarding risks. Each of these factors can amplify the potential for spillover of zoonotic pathogens, especially in contexts where knowledge is limited and/or some mitigation strategies are inaccessible.

Many respondents reported hunting species that are widely recognised as high-risk reservoirs of zoonotic pathogens, particularly bats, rodents, and primates. Bats have been repeatedly implicated in the transmission of viruses such as Ebola, Nipah, and coronaviruses [14,55–69], and their role as reservoirs for a wide range of viruses is well-established [70,71]. Similarly, primates are known carriers of various zoonotic agents, including simian immunodeficiency viruses, herpes B, Ebola, Marburg, rabies, and monkeypox [72–75]. Though transmission to humans remains relatively rare, the consequences can be severe, particularly given the high frequency of contact with rodents, which are known reservoirs for several zoonotic pathogens, reinforcing the need for cautious interaction and clear public health messaging.

Most respondents directly interacted with animal blood during hunting and meat preparation. This includes skinning, cutting, and eviscerating wild animals without protective barriers. Such contact is a recognised route for the transmission of many pathogens [76]. Injuries during meat handling further compound the risk. Our data indicate that over half of hunters and food preparers had experienced cuts or wounds while handling wild meat, increasing the likelihood of cross-species pathogen transmission via open skin lesions.

The absence of any form of PPE during the handling of wild meat remains widespread. Without gloves, aprons, or face coverings, exposure to infectious fluids is virtually unimpeded. Blood and tissue fluids may enter the body through mucous membranes, broken skin, or contaminated tools. In addition, respondents reported butchering meat on unsensitized wooden planks, a practice that promotes bacterial persistence and cross-contamination due to the porous nature of wood. Transitioning to non-porous, disinfectable surfaces and reinforcing hygienic handling practices are critical steps for public health protection [29].

Handling or consuming animals found dead in the forest poses significant risks, particularly when the cause of death is unknown. Scavenging carcasses may expose individuals to pathogens from animals that died of infectious diseases, as well as to the dangers of consuming spoiled meat. This practice was reported across several communities. Observations during fieldwork suggest that such behaviour is often driven by food insecurity or perceived economic necessity.

Evisceration of hunted animals, particularly rodents and bats, often occurs in domestic settings without basic sanitation. Several zoonotic pathogens, including Leptospira spp., hantaviruses, and filoviruses like the Ebola virus, can be transmitted to humans through direct contact with infected animals’ blood, organs, or other bodily fluids [77]. Women are especially vulnerable in these contexts because they are most involved in food preparation. Additionally, inspection of animals and meat is often cursory. About half of the respondents did not check animal flesh for anomalies, and a third admitted to removing visible lesions but consuming the remaining meat—a dangerous practice when there is no adequate training to identify a systemic infection [78].

Behaviours associated with zoonotic exposure varied across gender, ethnicity, and age groups. Indigenous Baka respondents were more likely than Bantu respondents to report hunting bats, a pattern that may increase their potential exposure to bat-hosted viruses. This difference likely reflects broader contrasts in subsistence practices, ecological knowledge, and hunting strategies between the two groups. Baka communities traditionally rely heavily on forest resources and exploit a wider diversity of wildlife species as part of their subsistence economy. Small-bodied species such as bats may be opportunistically captured during hunting trips or collected at known roosting sites. In contrast, Bantu hunters tend to focus more on larger mammals, typically using firearms or snares.

At the same time, some practices reported by Baka respondents may reduce exposure risk. For example, Baka participants were more likely than Bantu to report leaving animals found dead in the forest rather than taking them home, thereby avoiding contact with potentially diseased carcasses. However, Baka respondents were also less likely to wash their hands with soap after handling wildlife, a pattern that appears to reflect limited disposable income to purchase hygiene products rather than cultural norms. Gender also influenced exposure patterns. Women generally bear greater exposure risks during carcass processing and meat preparation due to gendered divisions of labour within households, which place them in frequent contact with animal tissues and bodily fluids.

Notably, concerns about disease risk appear to mediate protective behaviour. While fewer than 15% of respondents expressed concern about contact with the blood of freshly killed animals, those who did were more likely to adopt mitigation strategies, such as handwashing with soap and avoiding dead animals. These findings align with prior research showing that risk perception is crucial in driving health behaviour [79]. The low overall concern rate could stem from several factors: lack of awareness, disbelief in scientific messaging, risk habituation, or cultural beliefs. Even when people are concerned, however, practical constraints can limit behavioural change [80,81].

### Towards reducing zoonotic disease risk: from knowledge to action

Providing information alone is rarely sufficient to change behaviour, particularly when risks are poorly perceived, viewed as abstract, or when safer practices are difficult to implement in daily life. This is especially true in contexts where livelihoods depend on activities such as hunting, butchering, and selling wild meat, and where hygienic infrastructure and protective resources are limited. In such settings, awareness of zoonotic disease risks may exist, yet economic necessity, cultural norms, and the absence of viable alternatives constrain the ability of individuals to act on this knowledge.

Effective interventions must therefore move beyond simple knowledge transmission and support sustained, community-driven behaviour change. Achieving this requires a deeper understanding of how people perceive, prioritise, and respond to health risks within their social, cultural, and economic environments. Rather than focusing solely on what individuals know, research and interventions should also examine how knowledge circulates within communities, how risks are framed and interpreted, and what practical options exist for reducing exposure.

Future research would benefit from integrated methodological approaches that combine quantitative surveys with qualitative and participatory methods. Ethnographic approaches—including long-term observation, informal conversations, and immersion in daily routines—can provide valuable insights into the cultural, social, and symbolic dimensions of hunting, meat handling, and disease risk perception. These methods can reveal local explanatory models of illness, social norms, and decision-making processes that shape behaviour. Tools such as social network analysis can help identify how information and influence circulate within communities, while behavioural experiments may help determine which communication strategies or incentives are most effective in encouraging safer practices. Although qualitative methods were conducted in parallel with this study, their findings are not presented here to maintain the focus on the quantitative analyses.

Beyond understanding risk perceptions, successful behaviour change also requires culturally appropriate communication strategies and the removal of structural barriers that limit the adoption of safer practices. Health messages that connect zoonotic risks to familiar experiences—such as previous local disease outbreaks—may be more effective than abstract warnings. Participatory engagement with local leaders, hunters, women’s groups, and other community actors can help ensure that interventions are trusted, context-appropriate, and responsive to local priorities.

Crucially, behaviour change is unlikely to occur where safer choices remain inaccessible. Limited access to affordable protective equipment, inadequate hygiene infrastructure, and weak veterinary and public health surveillance systems all reduce the feasibility of adopting risk-reducing behaviours. Interventions must therefore be accompanied by structural investments in potable water, improved hygiene infrastructure, training in safe handling practices, and strengthened veterinary and human health services. Empowering communities to make safer choices requires not only knowledge but also the material means to act on it.

Our findings further highlight the importance of tailoring interventions to different socio-cultural and demographic contexts. For example, Indigenous Baka respondents were more likely to engage in certain practices that may increase exposure to zoonotic pathogens, while being less likely to engage in others, such as handling animals found dead. These patterns suggest that Indigenous and marginalised groups may require targeted outreach and support that recognises both their vulnerabilities and their existing risk-reduction practices. Gender differences in exposure, particularly during meat preparation due to the gendered division of labour, further emphasise the importance of inclusive strategies that recognise the distinct roles and risks faced by women within wild meat systems.

Reducing zoonotic disease risks in wild meat systems ultimately requires an integrated One Health approach that recognises the interconnectedness of human, animal, and environmental health. Cross-sector collaboration involving public health authorities, conservation organisations, veterinary services, and social scientists will be essential for designing effective interventions. Embedding zoonotic risk reduction within broader development, conservation, and livelihood programmes—while strengthening local surveillance and response systems—can help ensure that interventions are both sustainable and locally relevant.

Finally, this study has several limitations. Participants were classified according to their primary role in the wild meat value chain (e.g., hunter, vendor, or food preparer), but in practice individuals may perform multiple roles within wildlife trade networks. Previous studies have identified additional actor categories, such as transporters and middlemen, that play important roles in wildlife supply chains (e.g., Saylors et al., 2021). Although our classification captured the main functional roles relevant to this analysis, future research should further explore the diversity of actor types and how their interactions influence patterns of disease risk along wildlife trade networks.

## Conclusion

This study provides critical insight into the behaviours that increase the risk of zoonotic disease transmission in communities involved in hunting, preparing, and trading wild meat. High levels of exposure to blood and carcasses, lack of protective equipment, domestic evisceration, and unsafe meat inspection practices were all found to be widespread. Furthermore, the perception of risk, particularly concern about contact with animal blood, was significantly associated with protective behaviours, suggesting that enhancing awareness remains a foundational component of any intervention.

However, knowledge alone will not lead to behaviour change unless people also believe that safer practices are feasible, beneficial, and supported by their communities. Moving from risk awareness to risk reduction will require education, structural change, and inclusive, community-led strategies. We can only hope to prevent future zoonotic disease outbreaks and protect both human and ecological health by bridging local knowledge, public health capacity, and socio-economic realities.

## Supporting information

S1 Table Hunter and Food Preparer Questionnaire. (PDF)

S2 Table Vendor Questionnaire. (PDF)

S3 Checklist National Committee for Health Research Ethics. (PDF)
