# Supplementary material for: Cultural and behavioural drivers of zoonotic disease risk along the wild meat value chain in rural Cameroon
Source: PLoS One. 2026 Aug 5;21(8):e0355275. doi: 10.1371/journal.pone.0355275 (PMC13440831; doi:10.1371/journal.pone.0355275)
Supplement: Supplementary file 1 [file pone.0355275.s001.pdf]

[Hunter Food Preparer Questionnaire]
Mitigating Risks of Disease Transmission in Wild Meat

BRIEF DESCRIPTION OF THE RESEARCH

MANY WILD SPECIES ARE WIDELY HUNTED AND PROVIDE IMPORTANT FOOD, NUTRITIONAL AND FINANCIAL BENEFITS. HOWEVER, WILD ANIMALS USED FOR FOOD ARE MAJOR RESERVOIRS FOR A NUMBER OF FOODBORNE PATHOGENS FROM BACTERIA AND PARASITES AS WELL AS VIRUSES (E.G. EBOLA, MARBURG AND OTHERS) TRANSMITTED FROM ANIMAL BODY FLUIDS. MANY ZOONOTIC DISEASES ALSO POSE RISKS OF EPIDEMICS AND PANDEMICS. DESPITE THE POTENTIAL DANGERS ASSOCIATED WITH WILDLIFE USE, THERE ARE PRACTICAL WAYS OF REDUCING THE RISK OF CONTAGION AT DIFFERENT POINTS IN THE WILD MEAT FOOD CHAIN. THE CENTER FOR INTERNATIONAL FORESTRY RESEARCH IN COLLABORATION WITH FOREST RESOURCES FOR PEOPLE, WITH FUNDING FROM GIZ, ARE CARRYING OUT A TWO-YEAR PROJECT IN CAMEROON TO UNDERSTAND THE BEHAVIORS THAT MAY EXPOSE ACTORS TO DIFFERENT RISKS ALONG THE WILD MEAT FOOD CHAIN FROM THE FOREST TO THE FORK. THE INTENDED OUTCOME OF THIS PROJECT IS TO RECOMMEND ACTIVITIES AND POLICIES THAT CAN REDUCE THE RISKS OF DISEASE TRANSMISSION.

ENTER A DATE

PLEASE REQUEST A CONSENT

please tick the checkbox after consent is sought

○ OK

INTERVIEWEE DETAILS (A-K)

A. INTERVIEWER NAME *

B. INTERVIEWEE ID NUMBER *

C. STUDY AREA *

D. LOCALITY *

E. GENDER *

- ◯ Male
- ◯ Female

F. WHAT IS YOUR PRIMARY OCCUPATION? *

tick only one option

- ◯ Farmer
- ◯ Hunter
- ◯ Fisher
- ◯ Collecting NTFP selling (excluding hunting and fishing)
- ◯ Home maker
- ◯ Pensioner/retired
- ◯ Teacher
- ◯ Other

PLEASE SPECIFY:

G. IS YOUR PRIMARY OCCUPATION PAID OR UNPAID?

- Paid
- Unpaid

H. IS YOUR PRIMARY OCCUPATION TEMPORARY OR PERMANENT?

- Temporary
- Permanent

I. WHAT IS YOUR SECONDARY OCCUPATION? *

tick only one option

- Farmer
- Hunter
- Fisher
- Collecting NTFP selling (excluding hunting and fishing)
- Home maker
- Pensioner/retired
- Teacher
- Other

PLEASE SPECIFY:

J. IS YOUR SECONDARY OCCUPATION PAID OR UNPAID?

- Paid
- Unpaid

K. IS YOUR SECONDARY OCCUPATION TEMPORARY OR PERMANENT?

- Temporary
- Permanent

HUNTING WILDLIFE (Q1 - Q16)

Q1. DO YOU EVER HUNT?

by hunt, we mean not only going into the bush, but also trapping, catching animals in your fields.

- ◯ Yes
- ◯ No

IF ANSWER NO, WHY NOT?

Q2. WITHIN THE PREVIOUS 30 DAYS, HOW MANY TIMES DID YOU HUNT, TRAP OR CATCH ANIMALS?

Q3. WITHIN THE PREVIOUS 30 DAYS, HOW MANY TIMES DID YOU FISH?

Q4. WHAT METHOD DO YOU USUALLY USE FOR HUNTING?

tick more than one box if necessary

- ☐ Shotgun
- ☐ Spear
- ☐ Bow and arrow
- ☐ Snares
- ☐ Dogs
- ☐ Nets
- ☐ Glue
- ☐ Machete
- ☐ Other

PLEASE SPECIFY:

Q5. WHY DO YOU HUNT?

you may choose more than one response

- to sell for income
- for food for family
- enjoy the experience
- cultural or religious reasons
- other

PLEASE SPECIFY:

Q6. PLEASE LIST THE FIVE (5) SPECIES THAT YOU SELL MOST OFTEN:

Q6A. SPECIES 1:

- 1. Athérure/Porc épic (Mboké/Ndjouh/Ngome)
- 2. Aulacode/hérisson (Pankomo/Mvep)
- 3. Bongo (Mbongo/Zono/nzono/Ezona
- 4. Buffle (Mboko/Zomo/nzomo/Nyat)
- 5. Calao à joues grises (Kata/Dam/Zanga miam)
- 6. Calao longibande (Pkwang/Opkwepkwae)
- 7. Céphalophe à dos jaune (Bemba/Edjem/Zip)
- 8. Céphalophe bleu/lièvre (Dengbwe/Kuya/Opkweng)
- 9. Cephalophe de Bate (Kou/koup/Odjoe)
- 10. Céphalophe de Peter/biche (Nguedi/Mpine/Mvine)
- 11. Céphalphe à bande dorsale noire/biche (Ngbwom/Etyéné/Sô)
- 12. Cercocèbe agile (Pêh/pôh/Nsak)
- 13. Cercopithèque de brazza (Tamba/Pouo/Foum)
- 14. Chat doré (Ebié/Ebio)
- 15. Chauve-souris (Lipkwombwolo/Pebe/ndjêêm/Onguem/otane)
- 16. Chevrotain aquatique (Akolo/Akolo/Vion)
- 17. Chimpanzé (Séko/Gbwah/Wo'o)
- 18. Colobe guerezza/magistrat (Kalou/Djane/Sossolo)
- 19. Crocodile (Moukakélé/Mbane/Okom ngane)
- 20. Daman d'arbre (Yoka/Nyo'o/Nyok)
- 21. Ecureuil (Mpouh/Mvok)
- 22. Eléphant (Ya/Voh/mzohZok)
- 23. Genette (Tyama/Sii/Nsing)
- 24. Gorille (Ebobo/Ntilé/Ndji)
- 25. Grand calao à casque noire/calao (Mango/Douo/Ongoung)
- 26. Hocheur/nez blanc (Keuêh/Avembe)
- 27. Hylochère/phacochère (Nkoh/Engak)
- 28. Mandrill/Cinocéphale (Tamba/Sièh/Sek)
- 29. Mangouste (Mpôh/Mvak)
- 30. Mangouste (Nyamessouh/Nyamesso'o)
- 31. Mécanicien (Kôh/Kak)
- 32. Mone (Piné/Essouma)
- 33. Moustac (Souh/Ossok)
- 34. Nandinie/Chat tigre (Mbonga/Mpah/Mvae)
- 35. Oryctérope/kangourou (Pkwingna/Biame/Mvom ila)
- 36. Pangolin (Kokolo/Ziel/nziel/Okekaa)
- 37. Pangolin (Kokolo/Zièel/nzièl/Okekaa)
- 38. Pangolin géant (Kélépa/Dumé/Avil/zoh ka)
- 39. Panthère (Soua/Nkeuh/Ze)
- 40. Pérroquet (Koukoulou/Kûh/Kôss)
- 41. Picatharte chauve du cameroun
- 42. Potamochère/sanglier (Pamé/Nkou dîh/nkou dûh/Ngoe afane)
- 43. Potamogal (Lindje/Foupep/Djess)
- 44. Potto de bosman (Katou/Dinou/Awoune)
- 45. Python/boa (Méké/Mpouam/Mvom)
- 46. Rat (Gbwe/Ntaa/Koe si)
- 47. Ratel
- 48. Sitatunga/gazelle (Limboulou/Mpoul/Emvoul)
- 49. Talapoin (Atouo/Aozem)
- 50. Tortue (Kounda/Koul/Koulou)
- 51. Varan/Mbambé/Nkom/Nka'a)

Q6B. SPECIES 2:

- 1. Athérure/Porc épic (Mboké/Ndjouh/Ngome)
- 2. Aulacode/hérisson (Pankomo/Mvep)
- 3. Bongo (Mbongo/Zono/nzono/Ezona
- 4. Buffle (Mboko/Zomo/nzomo/Nyat)
- 5. Calao à joues grises (Kata/Dam/Zanga miam)
- 6. Calao longibande (Pkwang/Opkwepkwae)
- 7. Céphalophe à dos jaune (Bemba/Edjem/Zip)
- 8. Céphalophe bleu/lièvre (Dengbwe/Kuya/Opkweng)
- 9. Cephalophe de Bate (Kou/koup/Odjoe)
- 10. Céphalophe de Peter/biche (Nguedi/Mpine/Mvine)
- 11. Céphalphe à bande dorsale noire/biche (Ngbwom/Etyéné/Sô)
- 12. Cercocèbe agile (Pêh/pôh/Nsak)
- 13. Cercopithèque de brazza (Tamba/Pouo/Foum)
- 14. Chat doré (Ebié/Ebio)
- 15. Chauve-souris (Lipkwombwolo/Pebe/ndjêêm/Onguem/otane)
- 16. Chevrotain aquatique (Akolo/Akolo/Vion)
- 17. Chimpanzé (Séko/Gbwah/Wo'o)
- 18. Colobe guerezza/magistrat (Kalou/Djane/Sossolo)
- 19. Crocodile (Moukakélé/Mbane/Okom ngane)
- 20. Daman d'arbre (Yoka/Nyo'o/Nyok)
- 21. Ecureuil (Mpouh/Mvok)
- 22. Eléphant (Ya/Voh/mzohZok)
- 23. Genette (Tyama/Sii/Nsing)
- 24. Gorille (Ebobo/Ntilé/Ndji)
- 25. Grand calao à casque noire/calao (Mango/Douo/Ongoung)
- 26. Hocheur/nez blanc (Keuêh/Avembe)
- 27. Hylochère/phacochère (Nkoh/Engak)
- 28. Mandrill/Cinocéphale (Tamba/Sièh/Sek)
- 29. Mangouste (Mpôh/Mvak)
- 30. Mangouste (Nyamessouh/Nyamesso'o)
- 31. Mécanicien (Kôh/Kak)
- 32. Mone (Piné/Essouma)
- 33. Moustac (Souh/Ossok)
- 34. Nandinie/Chat tigre (Mbonga/Mpah/Mvae)
- 35. Oryctérope/kangourou (Pkwingna/Biame/Mvom ila)
- 36. Pangolin (Kokolo/Ziel/nziel/Okekaa)
- 37. Pangolin (Kokolo/Zièel/nzièl/Okekaa)
- 38. Pangolin géant (Kélépa/Dumé/Avil/zoh ka)
- 39. Panthère (Soua/Nkeuh/Ze)
- 40. Pérroquet (Koukoulou/Kûh/Kôss)
- 41. Picatharte chauve du cameroun
- 42. Potamochère/sanglier (Pamé/Nkou dîh/nkou dûh/Ngoe afane)
- 43. Potamogal (Lindje/Foupep/Djess)
- 44. Potto de bosman (Katou/Dinou/Awoune)
- 45. Python/boa (Méké/Mpouam/Mvom)
- 46. Rat (Gbwe/Ntaa/Koe si)
- 47. Ratel
- 48. Sitatunga/gazelle (Limboulou/Mpoul/Emvoul)
- 49. Talapoin (Atouo/Aozem)
- 50. Tortue (Kounda/Koul/Koulou)
- 51. Varan/Mbambé/Nkom/Nka'a)

Q6C. SPECIES 3:

- 1. Athérure/Porc épic (Mboké/Ndjouh/Ngome)
- 2. Aulacode/hérisson (Pankomo/Mvep)
- 3. Bongo (Mbongo/Zono/nzono/Ezona
- 4. Buffle (Mboko/Zomo/nzomo/Nyat)
- 5. Calao à joues grises (Kata/Dam/Zanga miam)
- 6. Calao longibande (Pkwang/Opkwepkwae)
- 7. Céphalophe à dos jaune (Bemba/Edjem/Zip)
- 8. Céphalophe bleu/lièvre (Dengbwe/Kuya/Opkweng)
- 9. Cephalophe de Bate (Kou/koup/Odjoe)
- 10. Céphalophe de Peter/biche (Nguedi/Mpine/Mvine)
- 11. Céphalphe à bande dorsale noire/biche (Ngbwom/Etyéné/Sô)
- 12. Cercocèbe agile (Pêh/pôh/Nsak)
- 13. Cercopithèque de brazza (Tamba/Pouo/Foum)
- 14. Chat doré (Ebié/Ebio)
- 15. Chauve-souris (Lipkwombwolo/Pebe/ndjêêm/Onguem/otane)
- 16. Chevrotain aquatique (Akolo/Akolo/Vion)
- 17. Chimpanzé (Séko/Gbwah/Wo'o)
- 18. Colobe guerezza/magistrat (Kalou/Djane/Sossolo)
- 19. Crocodile (Moukakélé/Mbane/Okom ngane)
- 20. Daman d'arbre (Yoka/Nyo'o/Nyok)
- 21. Ecureuil (Mpouh/Mvok)
- 22. Eléphant (Ya/Voh/mzohZok)
- 23. Genette (Tyama/Sii/Nsing)
- 24. Gorille (Ebobo/Ntilé/Ndji)
- 25. Grand calao à casque noire/calao (Mango/Douo/Ongoung)
- 26. Hocheur/nez blanc (Keuêh/Avembe)
- 27. Hylochère/phacochère (Nkoh/Engak)
- 28. Mandrill/Cinocéphale (Tamba/Sièh/Sek)
- 29. Mangouste (Mpôh/Mvak)
- 30. Mangouste (Nyamessouh/Nyamesso'o)
- 31. Mécanicien (Kôh/Kak)
- 32. Mone (Piné/Essouma)
- 33. Moustac (Souh/Ossok)
- 34. Nandinie/Chat tigre (Mbonga/Mpah/Mvae)
- 35. Oryctérope/kangourou (Pkwingna/Biame/Mvom ila)
- 36. Pangolin (Kokolo/Ziel/nziel/Okekaa)
- 37. Pangolin (Kokolo/Zièel/nzièl/Okekaa)
- 38. Pangolin géant (Kélépa/Dumé/Avil/zoh ka)
- 39. Panthère (Soua/Nkeuh/Ze)
- 40. Pérroquet (Koukoulou/Kûh/Kôss)
- 41. Picatharte chauve du cameroun
- 42. Potamochère/sanglier (Pamé/Nkou dîh/nkou dûh/Ngoe afane)
- 43. Potamogal (Lindje/Foupep/Djess)
- 44. Potto de bosman (Katou/Dinou/Awoune)
- 45. Python/boa (Méké/Mpouam/Mvom)
- 46. Rat (Gbwe/Ntaa/Koe si)
- 47. Ratel
- 48. Sitatunga/gazelle (Limboulou/Mpoul/Emvoul)
- 49. Talapoin (Atouo/Aozem)
- 50. Tortue (Kounda/Koul/Koulou)
- 51. Varan/Mbambé/Nkom/Nka'a)

Q6D. SPECIES 4:

- 1. Athérure/Porc épic (Mboké/Ndjouh/Ngome)
- 2. Aulacode/hérisson (Pankomo/Mvep)
- 3. Bongo (Mbongo/Zono/nzono/Ezona
- 4. Buffle (Mboko/Zomo/nzomo/Nyat)
- 5. Calao à joues grises (Kata/Dam/Zanga miam)
- 6. Calao longibande (Pkwang/Opkwepkwae)
- 7. Céphalophe à dos jaune (Bemba/Edjem/Zip)
- 8. Céphalophe bleu/lièvre (Dengbwe/Kuya/Opkweng)
- 9. Cephalophe de Bate (Kou/koup/Odjoe)
- 10. Céphalophe de Peter/biche (Nguedi/Mpine/Mvine)
- 11. Céphalphe à bande dorsale noire/biche (Ngbwom/Etyéné/Sô)
- 12. Cercocèbe agile (Pêh/pôh/Nsak)
- 13. Cercopithèque de brazza (Tamba/Pouo/Foum)
- 14. Chat doré (Ebié/Ebio)
- 15. Chauve-souris (Lipkwombwolo/Pebe/ndjêêm/Onguem/otane)
- 16. Chevrotain aquatique (Akolo/Akolo/Vion)
- 17. Chimpanzé (Séko/Gbwah/Wo'o)
- 18. Colobe guerezza/magistrat (Kalou/Djane/Sossolo)
- 19. Crocodile (Moukakélé/Mbane/Okom ngane)
- 20. Daman d'arbre (Yoka/Nyo'o/Nyok)
- 21. Ecureuil (Mpouh/Mvok)
- 22. Eléphant (Ya/Voh/mzohZok)
- 23. Genette (Tyama/Sii/Nsing)
- 24. Gorille (Ebobo/Ntilé/Ndji)
- 25. Grand calao à casque noire/calao (Mango/Douo/Ongoung)
- 26. Hocheur/nez blanc (Keuêh/Avembe)
- 27. Hylochère/phacochère (Nkoh/Engak)
- 28. Mandrill/Cinocéphale (Tamba/Sièh/Sek)
- 29. Mangouste (Mpôh/Mvak)
- 30. Mangouste (Nyamessouh/Nyamesso'o)
- 31. Mécanicien (Kôh/Kak)
- 32. Mone (Piné/Essouma)
- 33. Moustac (Souh/Ossok)
- 34. Nandinie/Chat tigre (Mbonga/Mpah/Mvae)
- 35. Oryctérope/kangourou (Pkwingna/Biame/Mvom ila)
- 36. Pangolin (Kokolo/Ziel/nziel/Okekaa)
- 37. Pangolin (Kokolo/Zièel/nzièl/Okekaa)
- 38. Pangolin géant (Kélépa/Dumé/Avil/zoh ka)
- 39. Panthère (Soua/Nkeuh/Ze)
- 40. Pérroquet (Koukoulou/Kûh/Kôss)
- 41. Picatharte chauve du cameroun
- 42. Potamochère/sanglier (Pamé/Nkou dîh/nkou dûh/Ngoe afane)
- 43. Potamogal (Lindje/Foupep/Djess)
- 44. Potto de bosman (Katou/Dinou/Awoune)
- 45. Python/boa (Méké/Mpouam/Mvom)
- 46. Rat (Gbwe/Ntaa/Koe si)
- 47. Ratel
- 48. Sitatunga/gazelle (Limboulou/Mpoul/Emvoul)
- 49. Talapoin (Atouo/Aozem)
- 50. Tortue (Kounda/Koul/Koulou)
- 51. Varan/Mbambé/Nkom/Nka'a)

Q6E. SPECIES 5:

- 1. Athérure/Porc épic (Mboké/Ndjouh/Ngome)
- 2. Aulacode/hérisson (Pankomo/Mvep)
- 3. Bongo (Mbongo/Zono/nzono/Ezona
- 4. Buffle (Mboko/Zomo/nzomo/Nyat)
- 5. Calao à joues grises (Kata/Dam/Zanga miam)
- 6. Calao longibande (Pkwang/Opkwepkwae)
- 7. Céphalophe à dos jaune (Bemba/Edjem/Zip)
- 8. Céphalophe bleu/lièvre (Dengbwe/Kuya/Opkweng)
- 9. Cephalophe de Bate (Kou/koup/Odjoe)
- 10. Céphalophe de Peter/biche (Nguedi/Mpine/Mvine)
- 11. Céphalphe à bande dorsale noire/biche (Ngbwom/Etyéné/Sô)
- 12. Cercocèbe agile (Pêh/pôh/Nsak)
- 13. Cercopithèque de brazza (Tamba/Pouo/Foum)
- 14. Chat doré (Ebié/Ebio)
- 15. Chauve-souris (Lipkwombwolo/Pebe/ndjêêm/Onguem/otane)
- 16. Chevrotain aquatique (Akolo/Akolo/Vion)
- 17. Chimpanzé (Séko/Gbwah/Wo'o)
- 18. Colobe guerezza/magistrat (Kalou/Djane/Sossolo)
- 19. Crocodile (Moukakélé/Mbane/Okom ngane)
- 20. Daman d'arbre (Yoka/Nyo'o/Nyok)
- 21. Ecureuil (Mpouh/Mvok)
- 22. Eléphant (Ya/Voh/mzohZok)
- 23. Genette (Tyama/Sii/Nsing)
- 24. Gorille (Ebobo/Ntilé/Ndji)
- 25. Grand calao à casque noire/calao (Mango/Douo/Ongoung)
- 26. Hocheur/nez blanc (Keuêh/Avembe)
- 27. Hylochère/phacochère (Nkoh/Engak)
- 28. Mandrill/Cinocéphale (Tamba/Sièh/Sek)
- 29. Mangouste (Mpôh/Mvak)
- 30. Mangouste (Nyamessouh/Nyamesso'o)
- 31. Mécanicien (Kôh/Kak)
- 32. Mone (Piné/Essouma)
- 33. Moustac (Souh/Ossok)
- 34. Nandinie/Chat tigre (Mbonga/Mpah/Mvae)
- 35. Oryctérope/kangourou (Pkwingna/Biame/Mvom ila)
- 36. Pangolin (Kokolo/Ziel/nziel/Okekaa)
- 37. Pangolin (Kokolo/Zièel/nzièl/Okekaa)
- 38. Pangolin géant (Kélépa/Dumé/Avil/zoh ka)
- 39. Panthère (Soua/Nkeuh/Ze)
- 40. Pérroquet (Koukoulou/Kûh/Kôss)
- 41. Picatharte chauve du cameroun
- 42. Potamochère/sanglier (Pamé/Nkou dîh/nkou dûh/Ngoe afane)
- 43. Potamogal (Lindje/Foupep/Djess)
- 44. Potto de bosman (Katou/Dinou/Awoune)
- 45. Python/boa (Méké/Mpouam/Mvom)
- 46. Rat (Gbwe/Ntaa/Koe si)
- 47. Ratel
- 48. Sitatunga/gazelle (Limboulou/Mpoul/Emvoul)
- 49. Talapoin (Atouo/Aozem)
- 50. Tortue (Kounda/Koul/Koulou)
- 51. Varan/Mbambé/Nkom/Nka'a)

Q7. DO YOU HUNT PRIMATES OR BATS OR RATS/SMALL RODENTS?	Always	Sometimes	Often	Rarely	Never
PRIMATES *	○	○	○	○	○
BATS *	○	○	○	○	○
RATS/SMALL RODENTS *	○	○	○	○	○

IF NEVER, PLEASE SPECIFY:

Q7A. [PRIMATES] PLEASE SPECIFY WHICH ARE THE MOST HUNTED SPECIES:

Q7B. [BATS] PLEASE SPECIFY WHICH ARE THE MOST HUNTED SPECIES :

Q7C. [RATS/SMALL RODENTS] PLEASE SPECIFY WHICH ARE THE MOST HUNTED SPECIES :

Q8. HOW MANY PEOPLE IN YOUR HOUSEHOLD HUNT?

Q9. IF YOU SELL WILD MEAT, WHO DO YOU SELL TO?

tick more than one box if necessary

- ☐ Trader from town
- ☐ Trader in the village
- ☐ Directly to the nearest town market
- ☐ Restaurant
- ☐ Neighbours (consumers)
- ☐ Other

PLEASE SPECIFY:

Q10. HAVE YOU SOMETIMES HUNTED ANIMALS THAT SHOW SIGNS OF DISEASE?

e.g. parasites or worms.

- Yes
- No

Q10A. IF YES, PLEASE DESCRIBE THE LAST TIME THIS OCCURRED. WHAT WAS THE DISEASE AND WHAT WERE THE CIRCUMSTANCES?

Q11. HAVE YOU SOMETIMES HUNTED ANIMALS THAT SHOW SIGNS OF INJURIES?

- Yes
- No

Q11A. IF YES, PLEASE DESCRIBE THE LAST TIME THIS OCCURRED. WHAT WAS THE INJURY AND WHAT WERE THE CIRCUMSTANCES?

Q12. HAVE YOU EVER FOUND DEAD ANIMALS WHEN HUNTING?

- Yes
- No

Q12A. IF YES, WHAT DID YOU DO WITH THEM?

- Cook and eat them in camp
- Take home to cook and eat later
- Leave where found
- Sell them
- Other

PLEASE SPECIFY:

Q13. IF YOU SOMETIMES EAT THEM, HOW DO YOU CHOOSE WHETHER TO EAT OR LEAVE THE ANIMAL?

Q14. HOW DO YOU NORMALLY SELLS WILD MEAT? *(name of species refers to your answers in Q6)*	live animal	field dressed	parts	whole (dead)
SPECIES 1	○	○	○	○
SPECIES 2	○	○	○	○
SPECIES 3	○	○	○	○
SPECIES 4	○	○	○	○
SPECIES 5	○	○	○	○

Q15. IN WHAT CONDITION DO YOU NORMALLY SELL WILD MEAT? *(name of species refers to your answers in Q6)*	raw	partially smoked	charred/dried	cooked
SPECIES 1	○	○	○	○
SPECIES 2	○	○	○	○
SPECIES 3	○	○	○	○
SPECIES 4	○	○	○	○
SPECIES 5	○	○	○	○

Q16. HAVE YOU EVER SUFFERED ANY INJURIES WHILE HANDLING WILDMEAT WHILE PREPARING?

e.g. from cutting tools o, please specify the injuries and from which animals:

- ○ Yes
- ○ No

Q16A. IF THERE ARE MANY INSTANCES, PLEASE SPECIFY THE INJURIES AND FROM WHICH ANIMALS:

if there are many instances, then please describe the two most serious incidents

....

HANDLING WILD MEAT (Q17 - Q25)

Q17. DO YOU HANDLE WILD MEAT CARCASSES? *

- Yes
- No

Q17A. IF YES, DO YOU HANDLE WILD MEAT IN THE FOLLOWING CIRCUMSTANCES:

tick more than one box, if necessary

- hunting
- transporting it from bush/field home
- transporting it to buyer
- cut it into pieces
- process (eg smoke, dry, etc.)
- cook it
- other

PLEASE SPECIFY:

Q18. DO YOU USE ANY PROTECTION TO HANDLE NEWLY DEAD ANIMALS? *

e.g. apron, chest robes, gloves

- Yes
- No

Q18A. IF YES, PLEASE DESCRIBE: *

Q19. ARE YOU CONCERNED ABOUT BEING IN CONTACT WITH THE BLOOD OF FRESHLY DEAD ANIMALS? *

- Yes
- No

Q19A. IF YES, PLEASE SPECIFY WHY:

Q19B. IF NO, PLEASE SPECIFY WHY:

Q20. DO YOU USUALLY WASH YOUR HANDS AFTER HANDLING DEAD ANIMALS? *

- Yes
- No

Q21. WHAT DO YOU USE TO WASH YOUR HANDS? *

- water
- water and soap
- Other

PLEASE SPECIFY:

Q22. HOW OFTEN DO YOUR CLOTHES GET BLOOD STAINED WHEN BUTCHERING ANIMALS? *

- Always
- Often
- Sometimes
- Rarely
- Never

SPECIFY WHICH ANIMALS: *

Q23. AT HOME, WHO BUTCHERS THE WILD MEAT CARCASS? *

tick more than one box, if necessary

- [ ] You
- [ ] Wife
- [ ] Husband
- [ ] Daughter
- [ ] Son
- [ ] Mother
- [ ] Mother-in-law
- [ ] Father
- [ ] Father-in-law
- [ ] Grandmother
- [ ] Grandfather
- [ ] Other

PLEASE SPECIFY:

....

Q24. IS THERE ANYTHING THAT IS COMMONLY DONE TO REDUCE RISKS FROM INJURY OR SICKNESS FROM BUTCHERING WILDMEAT? *

please describe

....

Q25. DO YOU REMOVE THE VISCERA OF ALL HUNTED ANIMALS BEFORE TAKING THE CARCASS HOME? *

- ( ) Yes
- ( ) No

Q25A. IF YOU DON'T, WHICH ANIMALS ARE NOT EVISCERATED, SPECIFY: *

....

Q26. AT HOME, WHO COOKS WILD MEAT? *

tick more than one box, if necessary

- [ ] You
- [ ] Wife
- [ ] Husband
- [ ] Daughter
- [ ] Son
- [ ] Mother
- [ ] Mother-in-law
- [ ] Father
- [ ] Father-in-law
- [ ] Grandmother
- [ ] Grandfather
- [ ] Other

PLEASE SPECIFY:

EATING WILD MEAT (Q27 - Q43)

Q27. HOW FREQUENTLY DO YOU EAT WILD MEAT IN THE RAINY SEASON? *

- ◯ Daily
- ◯ Weekly
- ◯ Monthly
- ◯ Every months
- ◯ Every six months
- ◯ Once a year
- ◯ Less than once a year
- ◯ Never

Q28. HOW FREQUENTLY DO YOU EAT WILD MEAT IN THE DRY SEASON? *

- Daily
- Weekly
- Monthly
- Every months
- Every six months
- Once a year
- Less than once a year
- Never

Q29. HOW FREQUENTLY DO YOU EAT FISH IN THE RAINY SEASON? *

- Daily
- Weekly
- Monthly
- Every months
- Every six months
- Once a year
- Less than once a year
- Never

Q30. HOW FREQUENTLY DO YOU EAT FISH IN THE DRY SEASON? *

- Daily
- Weekly
- Monthly
- Every months
- Every six months
- Once a year
- Less than once a year
- Never

Q31. PLEASE LIST THE FIVE (5) SPECIES OF WILD MEAT THAT YOU CONSUME MOST OFTEN:

Q31A. SPECIES 1: *

- 1. Athérure/Porc épic (Mboké/Ndjouh/Ngome)
- 2. Aulacode/hérisson (Pankomo/Mvep)
- 3. Bongo (Mbongo/Zono/nzono/Ezona
- 4. Buffle (Mboko/Zomo/nzomo/Nyat)
- 5. Calao à joues grises (Kata/Dam/Zanga miam)
- 6. Calao longibande (Pkwang/Opkwepkwae)
- 7. Céphalophe à dos jaune (Bemba/Edjem/Zip)
- 8. Céphalophe bleu/lièvre (Dengbwe/Kuya/Opkweng)

Q31B. SPECIES 2: *

- 1. Athérure/Porc épic (Mboké/Ndjouh/Ngome)
- 2. Aulacode/hérisson (Pankomo/Mvep)
- 3. Bongo (Mbongo/Zono/nzono/Ezona
- 4. Buffle (Mboko/Zomo/nzomo/Nyat)
- 5. Calao à joues grises (Kata/Dam/Zanga miam)
- 6. Calao longibande (Pkwang/Opkwepkwae)
- 7. Céphalophe à dos jaune (Bemba/Edjem/Zip)
- 8. Céphalophe bleu/lièvre (Dengbwe/Kuya/Opkweng)

Q31C. SPECIES 3: *

- 1. Athérure/Porc épic (Mboké/Ndjouh/Ngome)
- 2. Aulacode/hérisson (Pankomo/Mvep)
- 3. Bongo (Mbongo/Zono/nzono/Ezona
- 4. Buffle (Mboko/Zomo/nzomo/Nyat)
- 5. Calao à joues grises (Kata/Dam/Zanga miam)
- 6. Calao longibande (Pkwang/Opkwepkwae)
- 7. Céphalophe à dos jaune (Bemba/Edjem/Zip)
- 8. Céphalophe bleu/lièvre (Dengbwe/Kuya/Opkweng)

Q31D. SPECIES 4: *

- 1. Athérure/Porc épic (Mboké/Ndjouh/Ngome)
- 2. Aulacode/hérisson (Pankomo/Mvep)
- 3. Bongo (Mbongo/Zono/nzono/Ezona
- 4. Buffle (Mboko/Zomo/nzomo/Nyat)
- 5. Calao à joues grises (Kata/Dam/Zanga miam)
- 6. Calao longibande (Pkwang/Opkwepkwae)
- 7. Céphalophe à dos jaune (Bemba/Edjem/Zip)
- 8. Céphalophe bleu/lièvre (Dengbwe/Kuya/Opkweng)

Q31E. SPECIES 5: *

- 1. Athérure/Porc épic (Mboké/Ndjouh/Ngome)
- 2. Aulacode/hérisson (Pankomo/Mvep)
- 3. Bongo (Mbongo/Zono/nzono/Ezona
- 4. Buffle (Mboko/Zomo/nzomo/Nyat)
- 5. Calao à joues grises (Kata/Dam/Zanga miam)
- 6. Calao longibande (Pkwang/Opkwepkwae)
- 7. Céphalophe à dos jaune (Bemba/Edjem/Zip)
- 8. Céphalophe bleu/lièvre (Dengbwe/Kuya/Opkweng)
- 9. Céphalophe de Baté (Kou/koup/Odjoe)
- 10. Céphalophe de Peter/biche (Nguedi/Mpine/Mvine)
- 11. Céphalophe à bande dorsale noire/biche (Ngbwom/Etyéné/Sô)
- 12. Cercocèbe agile (Peh/pôh/Nsak)
- 13. Cercopithèque de brazza (Tamba/Pouo/Foum)
- 14. Chat doré (Ebié/Ebio)
- 15. Chauve-souris (Lipkwombwolo/Pebe/ndjêêm/Onguem/otane)
- 16. Chevrotain aquatique (Akolo/Akolo/Vion)
- 17. Chimpanzé (Séko/Gbwah/Wo'o)
- 18. Colobe guerezza/magistrat (Kalou/Djane/Sossolo)
- 19. Crocodile (Moukakélé/Mbane/Okom ngane)
- 20. Daman d'arbre (Yoka/Nyo'o/Nyok)
- 21. Ecureuil (Mpouh/Mvok)
- 22. Eléphant (Ya/Voh/mzohZok)
- 23. Genette (Tyama/Sii/Nsing)
- 24. Gorille (Ebobo/Ntilé/Ndji)

Q32. HOW MANY TIMES IN A WEEK/MONTH DO YOU USUALLY EAT EACH OF THESE SPECIES

5 most consumed species (refers to your answers in Q31)

	every day	5-6x per week	3-4x per week	1-2x per week	2x per months	1x per months	every 2-3 months	every 6 months	1x a year
SPECIES 1	○	○	○	○	○	○	○	○	○
SPECIES 2	○	○	○	○	○	○	○	○	○

SPECIES 3

SPECIES 4

SPECIES 5

- 25. Grand calao à casque noire/calao (M..go/Dou../Ongou..g)
- 26. H..cheur/n.. blanc (..euêh/A...mbe)
- 27. Hylochère/phacochère (Nkoh/Engak)
- 28. Mandrill/Cinocéphale (Tamba/Sièh/Sek)
- 29. Mangouste (Mpôh/Mvak)
- 30. Mangouste (Nyamessoun/Nyamesso'o)
- 31. Mécanicien (Kôh/Kak)
- 32. Mone (Piné/Essouma)
- 33. Moustac (Souh/Ossok)
- 34. Nandinie/Chat tigre (Mbonga/Mpah/Mvae)
- 35. Oryctérope/kangourou (Pkwingna/Biame/Mvom ila)
- 36. Pangolin (Kokolo/Ziel/nziel/Okekaa)
- 37. Pangolin (Kokolo/Zièel/nzièl/Okekaa)
- 38. Pangolin géant (Kélépa/Dumé/Avil/zoh ka)
- 39. Panthère (Soua/Nkeuh/Ze)
- 40. Pérroquet (Koukoulou/Kûh/Kôss)
- 41. Picatharte chauve du cameroun
- 42. Potamochère/sanglier (Pamé/Nkou dîh/nkou dûh/Ngoe afane)
- 43. Potamogal (Lindje/Foupep/Djess)
- 44. Potto de bosman (Katou/Dinou/Awoune)
- 45. Python/boa (Méké/Mpouam/Mvom)
- 46. Rat (Gbwe/Ntaa/Koe si)
- 47. Ratel
- 48. Sitatunga/gazelle (Limboulou/Mpoul/Emvoul)
- 49. Talapoin (Atouo/Aozem)
- 50. Tortue (Kounda/Koul/Koulou)
- 51. Varan/Mbambé/Nkom/Nka'a)

Q33. WHAT VISCERA DO YOU USUALLY EAT FROM THE (5) MOST CONSUMED ANIMALS?

5 most consumed species (refers to your answers in Q31)

VISCERA DO YOU USUALLY EAT - SPECIES 1:

(name of species refers to your answers in Q31)

- [ ] Liver
- [ ] Lung
- [ ] Heart
- [ ] Kidneys
- [ ] Spleen
- [ ] Testicles
- [ ] Stomach
- [ ] Intestines
- [ ] None

VISCERA DO YOU USUALLY EAT - SPECIES 2:
(name of species refers to your answers in Q31)

- [ ] Liver
- [ ] Lung
- [ ] Heart
- [ ] Kidneys
- [ ] Spleen
- [ ] Testicles
- [ ] Stomach
- [ ] Intestines
- [ ] None

VISCERA DO YOU USUALLY EAT - SPECIES 3:
(name of species refers to your answers in Q31)

- [ ] Liver
- [ ] Lung
- [ ] Heart
- [ ] Kidneys
- [ ] Spleen
- [ ] Testicles
- [ ] Stomach
- [ ] Intestines
- [ ] None

VISCERA DO YOU USUALLY EAT - SPECIES 4:
(name of species refers to your answers in Q31)

- [ ] Liver
- [ ] Lung
- [ ] Heart
- [ ] Kidneys
- [ ] Spleen
- [ ] Testicles
- [ ] Stomach
- [ ] Intestines
- [ ] None

VISCERA DO YOU USUALLY EAT - SPECIES 5:
(name of species refers to your answers in Q31)

- [ ] Liver
- [ ] Lung
- [ ] Heart
- [ ] Kidneys
- [ ] Spleen
- [ ] Testicles
- [ ] Stomach
- [ ] Intestines
- [ ] None

Q34. DO YOU EAT RAW MEAT? *

- ◯ Daily
- ◯ Weekly
- ◯ Monthly
- ◯ Every months
- ◯ Every 6 months
- ◯ Once a year
- ◯ Less than once a year
- ◯ Never

Q34A. IF YES, SPECIFY WHICH ANIMALS: *

....

Q35. WHERE DO YOU USUALLY REMOVE THE VISCERA OF HUNTED ANIMALS? *

- ◯ In the same place where the animal is hunted
- ◯ In the house where I live
- ◯ Where I hunt, in a temporary shelter or camp
- ◯ Other

PLEASE SPECIFY: *

....

Q35A. WHEN YOU REMOVE THE VISCERA OF THE ANIMAL, WHAT DO YOU USUALLY DO WITH THEM? *

- ◯ I usually eat them
- ◯ I give them to my children
- ◯ I give them to my dogs
- ◯ I give them to my cats
- ◯ I give them to my chickens
- ◯ Throw them in the bush
- ◯ Throw them away
- ◯ Other

PLEASE SPECIFY: *

Q35B. WHERE ARE THEY THROWN AWAY? *

Q36. HOW DO YOU USUALLY COOK WILD MEAT AT HOME? *

tick all that apply

- ☐ Boiled in soup/stew
- ☐ Cooked over open fire
- ☐ Other

PLEASE SPECIFY:

Q37. WHEN YOU PREPARE MEAT, DO YOU CHECK THE ANIMAL'S FLESH AND ORGANS FOR INJURY, ABNORMALITIES, SIGNS OF DISEASE OR PARASITES? *

- ◯ Always
- ◯ Often
- ◯ Sometimes
- ◯ Rarely
- ◯ Never

Q37A. IF ALWAYS, OFTEN OR SOMETIMES, WHAT DO YOU LOOK FOR WHEN CHECKING?

Q38. WHAT DO YOU DO WHEN YOU FIND VISCERA WITH LESIONS? *

- Eat the viscera normally
- Eat the viscera whole but well cooked
- Remove the lesion and eat the rest
- Burn it
- Bury it
- Sell it
- Give to dog, cat or chickens
- Throw it in the bush
- Other

PLEASE SPECIFY:

Q39. DO YOU FIND LEAD PELLETS IN HUNTED ANIMALS WHEN PREPARING THEIR MEAT? *

- Always
- Often
- Sometimes
- Rarely
- Never

Q40. DO YOU REMOVE THE PELLETS FROM WILD MEAT BEFORE COOKING? *

- Always
- Often
- Sometimes
- Rarely
- Never

Q41. WHAT SURFACE DO YOU USE TO CUT FRESH MEAT?

Q42. DO YOU WASH THE SURFACE AFTER USING? *

- Always
- Often
- Sometimes
- Rarely
- Never

Q43. WHEN WASHING SURFACE DO YOU USE SOAP OR WATER? *

- water
- Soap and water
- Other

PLEASE SPECIFY:

OTHER POSSIBLE SOURCES OF INFECTION (Q44 - Q47)

Q44. WHICH TYPES OF LIVESTOCK DO YOU OWN? *

- Goats
- Sheep
- Cattle
- Chicken
- Pigs
- Nothing
- Other

PLEASE SPECIFY: *

Q45. DO YOU HAVE DOGS AT HOME? *

- Yes
- No

Q46. WHEN WAS THE LAST TIME MEMBERS OF YOUR FAMILY RECEIVED DEWORMING TREATMENT?

- in the last one month
- in the last 3 months
- in the last 6 months
- in the last year
- in the last 3 years
- never

Q47. DO YOU DEWORM YOUR DOGS?

- Yes
- No

EVIDENCE OF FOOD BORNE ILLNESS IN THE FAMILY (Q48 - Q50)

Q48. DO YOU KNOW OF ANY ILLNESSES THAT SOMEONE IN YOUR HOUSEHOLD HAS SUFFERED AFTER EATING MEAT OR VISCERA FROM WILD ANIMALS?

- Yes
- No

Q49. DO YOU KNOW WHAT DISEASE IT WAS? COULD YOU NAME IT?

Q50. CAN YOU DESCRIBE THEIR SYMPTOMS?

WATER USAGE (Q51)

Q51. WHERE DO YOU USUALLY OBTAIN WATER FOR DRINKING? *

- River/stream
- House Pump
- Shared Pump
- Tap (mains water supply)
- House borehole
- Other

INTERVIEWEE DETAILS (L-O)

L. AGE (APPROXIMATE IN YEARS)

- 18-25 years
- 26-40 years
- 41-60 years
- >60 years

M. FIRST NAME: *

N. ETHNICITY: *

- ◯ Nzime
- ◯ Bulu
- ◯ Badjoue
- ◯ Baka
- ◯ Fang
- ◯ Konabembe
- ◯ Kako
- ◯ Mvonvon
- ◯ Mbemo
- ◯ Other

PLEASE SPECIFY:

O. HOW MANY PEOPLE LIVE IN YOUR HOUSEHOLD (PEOPLE WHO GENERALLY EAT FROM THE SAME POT)?

THANK YOU VERY MUCH FOR YOUR TIME!
