# Supplementary material for: Cultural and behavioural drivers of zoonotic disease risk along the wild meat value chain in rural Cameroon
Source: PLoS One. 2026 Aug 5;21(8):e0355275. doi: 10.1371/journal.pone.0355275 (PMC13440831; doi:10.1371/journal.pone.0355275)
Supplement: Supplementary file 2 [file pone.0355275.s002.pdf]

Vendor QUESTIONNAIRE
Mitigating Risks of Disease Transmission in Wild Meat

Brief description of the research:

Many wild species are widely hunted and provide important food, nutritional and financial benefits. However, wild animals used for food are major reservoirs for a number of foodborne pathogens from bacteria and parasites as well as viruses (e.g. Ebola, Marburg and others) transmitted from animal body fluids. Many zoonotic diseases also pose risks of epidemics and pandemics. Despite the potential dangers associated with wildlife use, there are practical ways of reducing the risk of contagion at different points in the wild meat food chain.

The Center for International Forestry Research in collaboration with Forest Resources for People, with funding from GIZ, are carrying out a two-year project in Cameroon to understand the behaviors that may expose actors to different risks along the wild meat food chain from the forest to the fork. The intended outcome of this project is to recommend activities and policies that can reduce the risks of disease transmission.

Ask participants for their consent to participate.

Objective of vendor survey:
To understand the health risks that vendors and their customers face from the sale of wild meat to propose mitigating risk strategies.

Contextual Elements

Observations and impressions of the interviewer to be filled in discreetly after the interview so that the vendor is not made uncomfortable

- *Description of the venue, noticeable elements*
- What animals are displayed (include number)?
- What is the condition of the animals/meat – e.g. live, fresh meat, dried, cooked, etc.
- Where are animals displayed – eg. Floor, table. Etc.
- Is the area clean?

- If area is not clean, please describe

Interviewee Details	
1. Interviewer Name	
2. Interviewee ID Number	
3. Study Area	
4. Market	
5. Gender	Male ( ) Female ( )
6. What types of animals are you selling today?	
7. What are the five types of animals that you sell most often?	1. 2. 3. 4. 5.
8. For each of the types of meat in #7, In what state do you most often receive?	1. live ( )/ freshly killed ( ) /partially smoked ( )/ fully smoked( )/ dried ( ), cooked ( )<br>*2.*<br>*.*<br>*In Kobo: will need five entries for this*
9. Do you normally cut up the animal? If no, proceed to Q 21	Yes ( )/ no ( )
10. If yes to #9 where do you butcher? Please describe	Free text
11. Are the tools or areas used cleaned before or after the animal is processed?	No ( )/yes ( )<br>If yes, please describe: (probe for materials used (e.g., soap), source of water, etc.)

12. if any parts are discarded, please describe which parts and where and how are these are disposed of?	Free form answer
13. Is there a designated area in the market for throwing rubbish including animal waste?	No ( )/yes (.). If yes, please describe
13. Have you ever been injured when cutting?	No( ) yes ( ) if yes, Please describe injury. (if more than once, describe most serious injury)
14. If yes, to 13, how did you treat?	
15. Do you do anything to protect yourself when butchering an animal carcass?	No ( )/yes ( )<br>If yes, please describe
16. Are you concerned about any risks to yourself from butchering the animal?	No ( )/yes ( )<br>If yes, please describe
17. Do you normally wash your hands before butchering the animal?	No ( )/ yes (.). If yes, probe for where _______<br>And with soap or other?
18. Is the surface where you cut the animal generally clean before you use?	No( )/yes( )
19. Do you wash the surface after you cut the meat?	No ( )/ yes ( ). If yes, probe if with soap or other?
20. Do you normally wash your hands after you cut the meat?	No ( )/ yes ( ). If yes, probe if with soap or other?
21. Do you have protective equipment/clothes that you wear when you are at work? If no, skip to 24	No( )/yes ( ).
22. Which equipment/clothes?	Gloves ( )/apron ( )/shoes( )/mask ( )
23. When do you use/wear items in 22?	Slaughter ( )/butcher( )/clean (.)/always at work (.)
24. Do you do anything to maintain the freshness of the meat that you sell or to protect it from pests?	No ( )/yes ( )<br>If yes, please describe
25. How do you generally store meat?	
26. Are there differences in storage for some types of meat?	No ( )/yes ( )<br>If yes, please describe
27. What do you do if you suspect that the meat has gone bad?	
28. Have you ever seen worms or other signs of illness in an animal that you butchered?	No ( )/yes ( )<br>If yes, please describe

29. What do you normally do if you see signs of illness?	
30. Do you think that there are health risks of eating animals with such illnesses?	No ( )/yes ( )<br>If yes, please describe
31. Do you think that there are any health risks that you or other vendors in the market face from selling wild meat?	No ( )/yes ( )<br>If yes, please describe
32. Do these risks differ for different types of animals?	No ( )/yes ( )<br>If yes, please describe
33. Is there anything that you can do to reduce these risks? Please describe.	
34. Is there anything that others can do to help you reduce the risks? Please describe.	
35. Have you or anyone you know in the market ever had a sickness that people do not usually get?	No ( )/yes ( )<br>If yes, please describe
36. Do you know of any diseases that people can get from wild animals?	No ( )/yes ( )<br>If yes, please describe
37. What can people do to reduce those risks?	
38. Has a government official inspected your stall in the last year? If no, end survey.	No ( )/yes ( )
39. From which department?	
40. What did they do?	

Thank you very much for your time!
