# Supplementary material for: Cultural and behavioural drivers of zoonotic disease risk along the wild meat value chain in rural Cameroon
Source: PLoS One. 2026 Aug 5;21(8):e0355275. doi: 10.1371/journal.pone.0355275 (PMC13440831; doi:10.1371/journal.pone.0355275)
Supplement: Supplementary file 3 [file pone.0355275.s003.pdf]

NATIONAL COMMITTEE FOR HEALTH RESEARCH ETHICS

Order No. 0977/A/MINSANTE/SESP/SG/DROS of 18 April 2012 establishing, organising and governing the operation of ethics committees for health research within the structures under the Ministry in charge of public health

No. 2022/08/1481 CE/CNERSH/SP　　Yaoundé, 23 August 2022

setcominae@gmail.com

ETHICAL CLEARANCE

The National Committee for Health Research Ethics (CNERSH), at its ordinary session of 23 August 2022, reviewed the research project entitled *"Wild Meat Handling Project: Mitigating Risks of Disease Transmission in the Wild Meat Food Chain in Cameroon"*, submitted by Dr. Richard EBA'A Atyi, Principal Investigator, Center for International Forestry Research (CIFOR).

The project is of significant scientific and social interest. The general objective of this study is to understand behaviours that may expose actors to various risks throughout the wild meat value chain, with the aim of developing interventions and recommendations to reduce health risks associated with wild meat handling. The study procedures are well-documented and clearly presented. The risks associated with the study are identified, along with measures to prevent and minimise them. The information sheet and informed consent form, in both French and English, are well developed and easy to understand. The measures taken to ensure the confidentiality of the data collected are outlined in the documentation. The investigators' CVs describe them as competent individuals capable of successfully carrying out this study.

For all these reasons, the National Committee for Health Research Ethics approves the implementation of the current version of the protocol for a duration of one year.

The investigators are responsible for strictly adhering to the approved protocol and must not make any amendments, however minor, without prior approval from CNERSH. The investigators are expected to cooperate with any monitoring visits from CNERSH during the implementation of the approved protocol. The final project report must be submitted to CNERSH and to the health authorities of Cameroon.

This ethical clearance may be revoked in the event of non-compliance with current regulations or the above-mentioned recommendations.

In witness whereof, this ethical clearance is issued to serve as legally appropriate.

Copies to:　　Interim Chair

- Ministry of Public Health (MINSANTE)　　**Dr. ABONG Bwemba Thérèse**

Note: This ethical clearance does not exempt you from obtaining the Administrative Research Authorisation (AAR), which is required to conduct this study within Cameroonian territory. This authorisation will be issued by the Ministry of Public Health.
