# Supplementary material for: Cultural and behavioural drivers of zoonotic disease risk along the wild meat value chain in rural Cameroon
Source: PLoS One. 2026 Aug 5;21(8):e0355275. doi: 10.1371/journal.pone.0355275 (PMC13440831; doi:10.1371/journal.pone.0355275)
Supplement: Supplementary file 4 [file pone.0355275.s004.docx]

**Responses to Reviewers’ comment on “Cultural and behavioural drivers of zoonotic disease risk along the wild meat value chain in rural Cameroon”**

**Reviewer #1:**

*General*

1. This is a very interesting study that provides critical insight into a suite of behaviours among local actors in the wild meat value chain that significantly increase the risk of zoonotic disease transmission. This study adds a lot to the knowledge base and will be very helpful in the design of interventions that try to reduce zoonotic disease risks in wild meat systems through an integrated One Health approach. I fully agree with the authors that moving from risk awareness to risk reduction in the global tropics and particularly in sub-Sahara Africa will require education, structural change, and inclusive community-led strategies.

R.	We sincerely thank the reviewer for their positive and encouraging assessment of our manuscript. We appreciate the recognition of the contribution this study makes to understanding behaviours within wild meat value chains that may increase the risk of zoonotic disease transmission.

We fully agree with the reviewer that effective risk reduction in wild meat systems requires moving beyond awareness alone and towards integrated approaches that combine education, structural improvements, and inclusive community-led strategies. These elements are central to the One Health perspective underpinning this work, and we are pleased that the reviewer considers the study useful for informing the design of such interventions.

We are grateful for the reviewer’s supportive comments and encouragement.

*Specific*

2. Abstract
    Line 43: “a higher percentage of Baka…”
    Comment: Baka are indigenous people, so please always specify “indigenous

Baka”

R.	Done. Lines 40-41.

**3.**	Lines 45 -47: “women and older individuals were less likely to hunt or handle clinically abnormal animals. However, women were more likely to take home carcasses found dead and were more likely to hunt rodents”

Comment: did both indigenous Baka and Bantu women show similar behaviour regarding hunting? From my experience with these communities which is also confirmed by other studies, indigenous Baka women take part in hunting by setting snares which is not the case with Bantu women.

R.	We have clarified this point in Lines 44-47.

**4.**	Pre-study engagement

Lines 253- 254: “Consultations were held with administrative authorities (including the Senior Divisional Officer and District Officer)”.

Comment: administratively, Cameroon has no districts. It is divided into regions, divisions and sub-divisions. Administrative authorities include governors at the head of regions, divisional officers at the head of divisions, and sub-divisional officers at the head of sub-divisions. Please correct accordingly in the text.

R.	Corrected. New text in Lines 206-209.

**5.**	Lines 255 – 256: “councils, and village chiefs. These meetings introduced the study, clarified its objectives, and obtained the necessary permissions”.

Comment: I am happy the authors followed the right procedure to collect their data and ethical approval, but the administrative authorities mentioned above are not the ones to issue authorisation for collecting research data in Cameroon. The authors first had to get a research permit from the ministry of scientific research and innovation authorising them collect any data in Cameroon and afterwards a permit from the ministry of wildlife and forestry to work in a protected area (Dja reserve). Did they obtain these permits, and can they share the permit numbers? This is to be sure your study meets all applicable standards for the ethics of experimentation and research integrity.

R.	We thank the reviewer for raising this important point regarding research authorisations in Cameroon. We confirm that the study complied with all national research permitting requirements. Prior to fieldwork, research authorisation was obtained from the Ministry of Scientific Research and Innovation (MINRESI), which grants permission for scientific research activities in Cameroon. In addition, a permit to conduct research activities within the Dja Faunal Reserve was obtained from the Ministry of Forestry and Wildlife (MINFOF). The consultations with local administrative authorities (including councils and village chiefs) mentioned in the manuscript refer to the customary local approval and community entry procedures that are required before conducting fieldwork in rural communities. These meetings were undertaken after the national-level permits had been secured and were intended to introduce the study, explain its objectives, and obtain community-level consent to work in the villages. To clarify this in the manuscript, we have revised the relevant section to explicitly indicate that national research permits were obtained from MINRESI and MINFOF prior to the consultations with local authorities. The permit reference numbers have also been included in the revised manuscript.

6.	Lines 406 -410: “As a token of appreciation, after completing their interview, all participants received a small gift—a set of two bars of soap valued at approximately 700 XAF (USD $1.16). While participants were not informed about the gift in advance, some may have heard about it from others”.
Comment: given that some participants may have heard in advance from others about the small gift offered and certainly the questions asked, how can you be sure this did not significantly influence their responses, and introduced bias into your study? Why not share some of the questions in the text to clarify the situation?

R.	We have modified the text to account for the reviewer’s comment, see Lines 373-382.

**7.**	Results
Line 420: “vendor counts in West Dja were based on data from 16 out of the 17 surveyed markets”.

Comment: under the vendor column of Table 1, do the numbers represent number of vendors/people interviewed or the number of surveyed markets? It’s unclear.

R.	The vendor counts in Table 1 represent the number of individual vendors interviewed (Lines 402-405).

**8.**	Discussion
Lines 754 - 755: “For instance, Baka respondents were more likely than Bantu to report hunting bats”

Comment: the authors should explain briefly why the indigenous Baka were more likely than Bantu to hunt bats. Was it because bats are culturally important to the Baka which would mean some cultural behaviours engage people in some high-risk practices or was it because bats were much easier to catch?

R.	Further explanation of this point has been included (Lines 702-720).

**Reviewer #2:**

*General*

**1.**	The manuscript is an interesting study with findings that will contribute to knowledge in the wildlife value chain and zoonotic pathogen spillover.

R.	We thank the reviewer for their positive assessment of the manuscript and for recognising the contribution of this study to understanding the wildlife value chain and the risk of zoonotic pathogen spillover. We appreciate the reviewer’s encouraging comments and are pleased that the findings are considered relevant to advancing knowledge in this area.

*Specific*

2. The authors should consider revising the title to accurately reflect the study's findings.

R.	Revised title*: Cultural and behavioural drivers of zoonotic disease risk along the wild meat value chain in rural Cameroon.*

3. The introduction is too lengthy, and the authors should make it more concise.

R. Done. Lines 70-131.

**4.**	The authors should specifically mention in the introduction the pathogens that spill over from wildlife to humans in Cameroon, rather than generalizing the situation in the Congo Basin.

R.	We have revised the Introduction to explicitly identify zoonotic pathogens known to occur in Cameroon that are associated with wildlife and wild meat handling, rather than referring more broadly to the Congo Basin. The revised text now highlights several documented examples, including filoviruses such as Ebola and Marburg carried by fruit bats, simian retroviruses in non-human primates related to HIV, and bacterial infections such as leptospirosis, salmonellosis, and plague associated with small mammals including rodents and civets. We also clarify that human exposure can occur during activities such as hunting, butchering, transport, and food preparation, when contact with animal blood, bodily fluids, or contaminated surfaces is common. These additions make the epidemiological context of zoonotic spillover risks in Cameroon more explicit and strengthen the rationale for examining behavioural practices along wild meat value chains in the study area. We thank the reviewer for prompting this clarification, which has improved the specificity and relevance of the Introduction.

**5.**	A limitation of this study is the classification of the wild meat value actors. A study by Saylors et al 2021 in Cameroon better classify the value chain actors as hunters, middlemen, and transporters.

R.	The revised manuscript now clarifies that participants were classified according to their primary role in the wild meat value chain (hunter, vendor, or food preparer) for the purposes of the analysis. However, we recognise that in practice individuals may perform multiple roles within wildlife trade networks, and that additional actor categories—such as transporters and middlemen—can play important roles in the movement and distribution of wild meat. We now explicitly note that this classification simplifies the complexity of real-world value chains and may not fully capture the diversity of actors and interactions that shape exposure pathways and disease risk. The revised text also highlights that future research should further examine the broader range of actor types and their interactions, building on previous work (e.g., Saylors et al., 2021), to better understand how zoonotic risks emerge and propagate along wildlife trade networks.

**6**.	The authors in lines 444-445 claim vendors consistently received wildlife from intermediaries. Please, who are these intermediaries? Be specific, are value chain actors also.

R.	In this study, intermediaries refer to value chain actors who purchase wild meat directly from hunters in villages and transport or redistribute it to market vendors, typically in nearby towns or roadside markets. These individuals are often involved in the collection, aggregation, and transport of wild meat, linking rural hunters with vendors and consumers further along the trade chain. In some cases, intermediaries may also be involved in small-scale trading themselves, operating informally within local market networks. To clarify this point, we have revised the relevant section of the manuscript (Lines 244-252) to explicitly state that intermediaries are also actors within the wild meat value chain, functioning primarily as collectors or transporters who move wildlife products from hunters to vendors and other markets. This clarification helps distinguish their role from that of hunters, vendors, and household food preparers, while acknowledging their importance in the movement of wild meat within local trade networks.

7.	Did the authors ask questions on the hunting method, and why was it not presented? Hunting is a major risk factor for a spillover event.

R.	Information on hunting methods was indeed collected during the surveys, as these practices can influence the frequency and type of human–wildlife contact and therefore affect potential exposure to zoonotic pathogens. However, hunting methods were not included in the present analysis because the primary objective of this study was to examine risk perceptions and hygiene-related behaviours along the wild meat value chain, rather than to analyse ecological or technical aspects of hunting practices. To avoid expanding the scope of the manuscript beyond its main research questions, the analysis focused on behavioural practices directly related to handling, preparation, and consumption of wild meat. To clarify this point, we have added new text in Lines 240-242. This addition explains why these data were collected but not presented. We agree with the reviewer that hunting practices are an important factor in zoonotic spillover risk, and we plan to examine these data in future analyses focusing specifically on hunting techniques and wildlife contact patterns.
