# Supplementary material for: Cultural and behavioural drivers of zoonotic disease risk along the wild meat value chain in rural Cameroon
Source: PLoS One. 2026 Aug 5;21(8):e0355275. doi: 10.1371/journal.pone.0355275 (PMC13440831; doi:10.1371/journal.pone.0355275)
Supplement: Peer review history

# Peer review: Decision Letter 0

**DOI:** 10.1371/journal.pone.0355275.r001

**Authors:** Clement Meseko

15 Oct 2025

PONE-D-25-28273

PLOS ONE Editorial

Cultural and Behavioural Dimensions of Disease Risk in the Wild Meat Trade of Rural Cameroon

PLOS ONE

Dear Dr. Fa,

Thank you for submitting your manuscript to PLOS ONE. After careful consideration, we feel that it has merit but does not fully meet PLOS ONE’s publication criteria as it currently stands. Therefore, we invite you to submit a revised version of the manuscript that addresses the points raised during the review process.

Please submit your revised manuscript by Nov 29 2025 11:59PM. If you will need more time than this to complete your revisions, please reply to this message or contact the journal office at plosone@plos.org. When you're ready to submit your revision, log on to https://www.editorialmanager.com/pone/ and select the 'Submissions Needing Revision' folder to locate your manuscript file.

Please include the following items when submitting your revised manuscript:

- A rebuttal letter that responds to each point raised by the academic editor and reviewer(s). You should upload this letter as a separate file labeled 'Response to Reviewers'.
- A marked-up copy of your manuscript that highlights changes made to the original version. You should upload this as a separate file labeled 'Revised Manuscript with Track Changes'.
- An unmarked version of your revised paper without tracked changes. You should upload this as a separate file labeled 'Manuscript'.

-->If you would like to make changes to your financial disclosure, please include your updated statement in your cover letter. Guidelines for resubmitting your figure files are available below the reviewer comments at the end of this letter.

If applicable, we recommend that you deposit your laboratory protocols in protocols.io to enhance the reproducibility of your results. Protocols.io assigns your protocol its own identifier (DOI) so that it can be cited independently in the future. For instructions see: https://journals.plos.org/plosone/s/submission-guidelines#loc-laboratory-protocols. Additionally, PLOS ONE offers an option for publishing peer-reviewed Lab Protocol articles, which describe protocols hosted on protocols.io. Read more information on sharing protocols at https://plos.org/protocols?utm_medium=editorial-email&utm_source=authorletters&utm_campaign=protocols.

We look forward to receiving your revised manuscript.

Kind regards,

Clement Adebajo Meseko, DVM, PhD

Academic Editor

PLOS ONE

Journal Requirements:

When submitting your revision, we need you to address these additional requirements.

1. Please ensure that your manuscript meets PLOS ONE's style requirements, including those for file naming. The PLOS ONE style templates can be found at

https://journals.plos.org/plosone/s/file?id=wjVg/PLOSOne_formatting_sample_main_body.pdf and

https://journals.plos.org/plosone/s/file?id=ba62/PLOSOne_formatting_sample_title_authors_affiliations.pdf.

2. Please include a complete copy of PLOS’ questionnaire on inclusivity in global research in your revised manuscript. Our policy for research in this area aims to improve transparency in the reporting of research performed outside of researchers’ own country or community. The policy applies to researchers who have travelled to a different country to conduct research, research with Indigenous populations or their lands, and research on cultural artefacts. The questionnaire can also be requested at the journal’s discretion for any other submissions, even if these conditions are not met.  Please find more information on the policy and a link to download a blank copy of the questionnaire here: https://journals.plos.org/plosone/s/best-practices-in-research-reporting. Please upload a completed version of your questionnaire as Supporting Information when you resubmit your manuscript.

3. We suggest you thoroughly copyedit your manuscript for language usage, spelling, and grammar. If you do not know anyone who can help you do this, you may wish to consider employing a professional scientific editing service.

The American Journal Experts (AJE) (https://www.aje.com/) is one such service that has extensive experience helping authors meet PLOS guidelines and can provide language editing, translation, manuscript formatting, and figure formatting to ensure your manuscript meets our submission guidelines. Please note that having the manuscript copyedited by AJE or any other editing services does not guarantee selection for peer review or acceptance for publication.

Upon resubmission, please provide the following:

The name of the colleague or the details of the professional service that edited your manuscript

A copy of your manuscript showing your changes by either highlighting them or using track changes (uploaded as a *supporting information* file)

A clean copy of the edited manuscript (uploaded as the new *manuscript* file).

4. Thank you for stating the following financial disclosure:

“German Agency for International Cooperation GmbH, Deutsche fur Internationale Zusammenarbeit (GIZ), Agreement number: 81279235; Project processing number:20.2256.4-002.00”

Please state what role the funders took in the study.  If the funders had no role, please state: "The funders had no role in study design, data collection and analysis, decision to publish, or preparation of the manuscript."

If this statement is not correct you must amend it as needed.

Please include this amended Role of Funder statement in your cover letter; we will change the online submission form on your behalf.

5. In this instance it seems there may be acceptable restrictions in place that prevent the public sharing of your minimal data. However, in line with our goal of ensuring long-term data availability to all interested researchers, PLOS’ Data Policy states that authors cannot be the sole named individuals responsible for ensuring data access (http://journals.plos.org/plosone/s/data-availability#loc-acceptable-data-sharing-methods).

Data requests to a non-author institutional point of contact, such as a data access or ethics committee, helps guarantee long term stability and availability of data. Providing interested researchers with a durable point of contact ensures data will be accessible even if an author changes email addresses, institutions, or becomes unavailable to answer requests.

Before we proceed with your manuscript, please also provide non-author contact information (phone/email/hyperlink) for a data access committee, ethics committee, or other institutional body to which data requests may be sent. If no institutional body is available to respond to requests for your minimal data, please consider if there any institutional representatives who did not collaborate in the study, and are not listed as authors on the manuscript, who would be able to hold the data and respond to external requests for data access? If so, please provide their contact information (i.e., email address). Please also provide details on how you will ensure persistent or long-term data storage and availability.

6. Thank you for stating the following in the Acknowledgments Section of your manuscript:

“This work was funded by the “Mitigating risks of disease trans-mission in the wild meat food chain from forest to fork in Cameroon” project (Agreement number: 81279235; Project processing number:20.2256.4-002.00), funded by the German Agency for International Cooperation GmbH, Deutsche fur Internationale Zusammenarbeit (GIZ) as support to the International Alliance against Health Risks in Wildlife Trade. “

We note that you have provided additional information within the Acknowledgements Section that is not currently declared in your Funding Statement. Please note that funding information should not appear in the Acknowledgments section or other areas of your manuscript. We will only publish funding information present in the Funding Statement section of the online submission form.

Please remove any funding-related text from the manuscript and let us know how you would like to update your Funding Statement. Currently, your Funding Statement reads as follows:

“German Agency for International Cooperation GmbH, Deutsche fur Internationale Zusammenarbeit (GIZ), Agreement number: 81279235; Project processing number:20.2256.4-002.00”

Please include your amended statements within your cover letter; we will change the online submission form on your behalf.

7. Please amend the manuscript submission data (via Edit Submission) to include author **Guillermo Ros Brull^.^**

8. We note that Figure 1 in your submission contain [map/satellite] images which may be copyrighted. All PLOS content is published under the Creative Commons Attribution License (CC BY 4.0), which means that the manuscript, images, and Supporting Information files will be freely available online, and any third party is permitted to access, download, copy, distribute, and use these materials in any way, even commercially, with proper attribution. For these reasons, we cannot publish previously copyrighted maps or satellite images created using proprietary data, such as Google software (Google Maps, Street View, and Earth). For more information, see our copyright guidelines: http://journals.plos.org/plosone/s/licenses-and-copyright.

We require you to either (1) present written permission from the copyright holder to publish these figures specifically under the CC BY 4.0 license, or (2) remove the figures from your submission:

a. You may seek permission from the original copyright holder of Figure 1 to publish the content specifically under the CC BY 4.0 license.

We recommend that you contact the original copyright holder with the Content Permission Form (http://journals.plos.org/plosone/s/file?id=7c09/content-permission-form.pdf) and the following text:

“I request permission for the open-access journal PLOS ONE to publish XXX under the Creative Commons Attribution License (CCAL) CC BY 4.0 (http://creativecommons.org/licenses/by/4.0/). Please be aware that this license allows unrestricted use and distribution, even commercially, by third parties. Please reply and provide explicit written permission to publish XXX under a CC BY license and complete the attached form.”

Please upload the completed Content Permission Form or other proof of granted permissions as an "Other" file with your submission.

In the figure caption of the copyrighted figure, please include the following text: “Reprinted from [ref] under a CC BY license, with permission from [name of publisher], original copyright [original copyright year].”

b. If you are unable to obtain permission from the original copyright holder to publish these figures under the CC BY 4.0 license or if the copyright holder’s requirements are incompatible with the CC BY 4.0 license, please either i) remove the figure or ii) supply a replacement figure that complies with the CC BY 4.0 license. Please check copyright information on all replacement figures and update the figure caption with source information. If applicable, please specify in the figure caption text when a figure is similar but not identical to the original image and is therefore for illustrative purposes only.

The following resources for replacing copyrighted map figures may be helpful:

USGS National Map Viewer (public domain): http://viewer.nationalmap.gov/viewer/

The Gateway to Astronaut Photography of Earth (public domain): http://eol.jsc.nasa.gov/sseop/clickmap/

Maps at the CIA (public domain): https://www.cia.gov/library/publications/the-world-factbook/index.html and https://www.cia.gov/library/publications/cia-maps-publications/index.html

NASA Earth Observatory (public domain): http://earthobservatory.nasa.gov/

Landsat: http://landsat.visibleearth.nasa.gov/

USGS EROS (Earth Resources Observatory and Science (EROS) Center) (public domain): http://eros.usgs.gov/#

Natural Earth (public domain): http://www.naturalearthdata.com/

9. If the reviewer comments include a recommendation to cite specific previously published works, please review and evaluate these publications to determine whether they are relevant and should be cited. There is no requirement to cite these works unless the editor has indicated otherwise.

10. Please review your reference list to ensure that it is complete and correct. If you have cited papers that have been retracted, please include the rationale for doing so in the manuscript text, or remove these references and replace them with relevant current references. Any changes to the reference list should be mentioned in the rebuttal letter that accompanies your revised manuscript. If you need to cite a retracted article, indicate the article’s retracted status in the References list and also include a citation and full reference for the retraction notice.

[Note: HTML markup is below. Please do not edit.]

Reviewers' comments:

Reviewer's Responses to Questions

**Comments to the Author**

1. Is the manuscript technically sound, and do the data support the conclusions?

The manuscript must describe a technically sound piece of scientific research with data that supports the conclusions. Experiments must have been conducted rigorously, with appropriate controls, replication, and sample sizes. The conclusions must be drawn appropriately based on the data presented.

Reviewer #1: Yes

Reviewer #2: Yes

**********

2. Has the statistical analysis been performed appropriately and rigorously?

Reviewer #1: Yes

Reviewer #2: Yes

**********

3. Have the authors made all data underlying the findings in their manuscript fully available?

The PLOS Data policy requires authors to make all data underlying the findings described in their manuscript fully available without restriction, with rare exception (please refer to the Data Availability Statement in the manuscript PDF file). The data should be provided as part of the manuscript or its supporting information, or deposited to a public repository. For example, in addition to summary statistics, the data points behind means, medians and variance measures should be available. If there are restrictions on publicly sharing data—e.g. participant privacy or use of data from a third party—those must be specified.

Reviewer #1: Yes

Reviewer #2: Yes

**********

4. Is the manuscript presented in an intelligible fashion and written in standard English?

PLOS ONE does not copyedit accepted manuscripts, so the language in submitted articles must be clear, correct, and unambiguous. Any typographical or grammatical errors should be corrected at revision, so please note any specific errors here.

Reviewer #1: Yes

Reviewer #2: Yes

**********

5. Review Comments to the Author

Please use the space provided to explain your answers to the questions above. You may also include additional comments for the author, including concerns about dual publication, research ethics, or publication ethics. (Please upload your review as an attachment if it exceeds 20,000 characters)

Reviewer #1: This is a very interesting study that provides critical insight into a suite of behaviours among local actors in the wild meat value chain that significantly increase the risk of zoonotic disease transmission. This study adds a lot to the knowledge base and will be very helpful in the design of interventions that try to reduce zoonotic disease risks in wild meat systems through an integrated One Health approach. I fully agree with the authors that moving from risk awareness to risk reduction in the global tropics and particularly in sub-Sahara Africa will require education, structural change, and inclusive community-led strategies.

I however have few remarks on some points that need further clarification. My comments below:

Abstract

Line 43: “a higher percentage of Baka…”

Comment: Baka are indigenous people, so please always specify “indigenous Baka”

Lines 45 -47: “women and older individuals were less likely to hunt or handle clinically abnormal animals. However, women were more likely to take home carcasses found dead and were more likely to hunt rodents”

Comment: did both indigenous Baka and Bantu women show similar behaviour regarding hunting? From my experience with these communities which is also confirmed by other studies, indigenous Baka women take part in hunting by setting snares which is not the case with Bantu women.

Pre-study engagement

Lines 253- 254: “Consultations were held with administrative authorities (including the Senior Divisional Officer and District Officer)”

Comment: administratively, Cameroon has no districts. It is divided into regions, divisions and sub-divisions. Administrative authorities include governors at the head of regions, divisional officers at the head of divisions, and sub-divisional officers at the head of sub-divisions. Please correct accordingly in the text.

Lines 255 – 256: “councils, and village chiefs. These meetings introduced the study, clarified its objectives, and obtained the necessary permissions”.

Comment: I am happy the authors followed the right procedure to collect their data and ethical approval, but the administrative authorities mentioned above are not the ones to issue authorisation for collecting research data in Cameroon. The authors first had to get a research permit from the ministry of scientific research and innovation authorising them collect any data in Cameroon and afterwards a permit from the ministry of wildlife and forestry to work in a protected area (Dja reserve). Did they obtain these permits, and can they share the permit numbers? This is to be sure your study meets all applicable standards for the ethics of experimentation and research integrity.

Lines 406 -410: “As a token of appreciation, after completing their interview, all participants received a small gift—a set of two bars of soap valued at approximately 700 XAF (USD $1.16). While participants were not informed about the gift in advance, some may have heard about it from others”.

Comment: given that some participants may have heard in advance from others about the small gift offered and certainly the questions asked, how can you be sure this did not significantly influence their responses, and introduced bias into your study? Why not share some of the questions in the text to clarify the situation?

Results

Line 420: “vendor counts in West Dja were based on data from 16 out of the 17 surveyed markets”.

Comment: under the vendor column of Table 1, do the numbers represent number of vendors/people interviewed or the number of surveyed markets? It’s unclear.

Discussion

Lines 754 - 755: “For instance, Baka respondents were more likely than Bantu to report hunting bats”

Comment: the authors should explain briefly why the indigenous Baka were more likely than Bantu to hunt bats. Was it because bats are culturally important to the Baka which would mean some cultural behaviours engage people in some high-risk practices or was it because bats were much easier to catch?

Reviewer #2: Dear Authors

The manuscript is an interesting study with findings that will contribute to knowledge in the wildlife value chain and zoonotic pathogen spillover.

However, I have the following comments for the authors.

1. The authors should consider revising the title to accurately reflect the study's findings.

2. “Cultural and Behavioural Dimensions of Disease Risk in the Wild Meat Value Chain Trade of Rural Cameroon”

3. The introduction is too lengthy, and the authors should make it more concise.

4. The authors should specifically mention in the introduction the pathogens that spill over from wildlife to humans in Cameroon, rather than generalizing the situation in the Congo Basin.

5. A limitation of this study is the classification of the wild meat value actors.

6. A study by Saylors et al 2021 in Cameroon better classify the value chain actors as hunters, middlemen, and transporters.

7. The authors in lines 444-445 claim vendors consistently received wildlife from intermediaries. Please, who are these intermediaries? Be specific, are value chain actors also

8. Did the authors ask questions on the hunting method, and why was it not presented ? Hunting is a major risk factor for a spillover event.

**********

6. PLOS authors have the option to publish the peer review history of their article (what does this mean?). If published, this will include your full peer review and any attached files.

If you choose “no”, your identity will remain anonymous but your review may still be made public.

**Do you want your identity to be public for this peer review?**  For information about this choice, including consent withdrawal, please see our Privacy Policy.

Reviewer #1: **Yes:** Eric Djomo Nana

Reviewer #2: **Yes:** Adeyinka Jeremy Adedeji

**********

[NOTE: If reviewer comments were submitted as an attachment file, they will be attached to this email and accessible via the submission site. Please log into your account, locate the manuscript record, and check for the action link "View Attachments". If this link does not appear, there are no attachment files.]

While revising your submission, please upload your figure files to the Preflight Analysis and Conversion Engine (PACE) digital diagnostic tool, https://pacev2.apexcovantage.com/. PACE helps ensure that figures meet PLOS requirements. To use PACE, you must first register as a user. Registration is free. Then, login and navigate to the UPLOAD tab, where you will find detailed instructions on how to use the tool. If you encounter any issues or have any questions when using PACE, please email PLOS at figures@plos.org. Please note that Supporting Information files do not need this step.

# Author comment: Author response to Decision Letter 1

**DOI:** 10.1371/journal.pone.0355275.r002

9 Apr 2026

Responses to Reviewers’ comment on “Cultural and behavioural drivers of zoonotic disease risk along the wild meat value chain in rural Cameroon”

Reviewer #1:

General

1. This is a very interesting study that provides critical insight into a suite of behaviours among local actors in the wild meat value chain that significantly increase the risk of zoonotic disease transmission. This study adds a lot to the knowledge base and will be very helpful in the design of interventions that try to reduce zoonotic disease risks in wild meat systems through an integrated One Health approach. I fully agree with the authors that moving from risk awareness to risk reduction in the global tropics and particularly in sub-Sahara Africa will require education, structural change, and inclusive community-led strategies.

R. We sincerely thank the reviewer for their positive and encouraging assessment of our manuscript. We appreciate the recognition of the contribution this study makes to understanding behaviours within wild meat value chains that may increase the risk of zoonotic disease transmission.

We fully agree with the reviewer that effective risk reduction in wild meat systems requires moving beyond awareness alone and towards integrated approaches that combine education, structural improvements, and inclusive community-led strategies. These elements are central to the One Health perspective underpinning this work, and we are pleased that the reviewer considers the study useful for informing the design of such interventions.

We are grateful for the reviewer’s supportive comments and encouragement.

Specific

2. Abstract

Line 43: “a higher percentage of Baka…”

Comment: Baka are indigenous people, so please always specify “indigenous

Baka”

R. Done. Lines 40-41.

3. Lines 45 -47: “women and older individuals were less likely to hunt or handle clinically abnormal animals. However, women were more likely to take home carcasses found dead and were more likely to hunt rodents”

Comment: did both indigenous Baka and Bantu women show similar behaviour regarding hunting? From my experience with these communities which is also confirmed by other studies, indigenous Baka women take part in hunting by setting snares which is not the case with Bantu women.

R. We have clarified this point in Lines 44-47.

4. Pre-study engagement

Lines 253- 254: “Consultations were held with administrative authorities (including the Senior Divisional Officer and District Officer)”.

Comment: administratively, Cameroon has no districts. It is divided into regions, divisions and sub-divisions. Administrative authorities include governors at the head of regions, divisional officers at the head of divisions, and sub-divisional officers at the head of sub-divisions. Please correct accordingly in the text.

R. Corrected. New text in Lines 206-209.

5. Lines 255 – 256: “councils, and village chiefs. These meetings introduced the study, clarified its objectives, and obtained the necessary permissions”.

Comment: I am happy the authors followed the right procedure to collect their data and ethical approval, but the administrative authorities mentioned above are not the ones to issue authorisation for collecting research data in Cameroon. The authors first had to get a research permit from the ministry of scientific research and innovation authorising them collect any data in Cameroon and afterwards a permit from the ministry of wildlife and forestry to work in a protected area (Dja reserve). Did they obtain these permits, and can they share the permit numbers? This is to be sure your study meets all applicable standards for the ethics of experimentation and research integrity.

R. We thank the reviewer for raising this important point regarding research authorisations in Cameroon. We confirm that the study complied with all national research permitting requirements. Prior to fieldwork, research authorisation was obtained from the Ministry of Scientific Research and Innovation (MINRESI), which grants permission for scientific research activities in Cameroon. In addition, a permit to conduct research activities within the Dja Faunal Reserve was obtained from the Ministry of Forestry and Wildlife (MINFOF). The consultations with local administrative authorities (including councils and village chiefs) mentioned in the manuscript refer to the customary local approval and community entry procedures that are required before conducting fieldwork in rural communities. These meetings were undertaken after the national-level permits had been secured and were intended to introduce the study, explain its objectives, and obtain community-level consent to work in the villages. To clarify this in the manuscript, we have revised the relevant section to explicitly indicate that national research permits were obtained from MINRESI and MINFOF prior to the consultations with local authorities. The permit reference numbers have also been included in the revised manuscript.

6. Lines 406 -410: “As a token of appreciation, after completing their interview, all participants received a small gift—a set of two bars of soap valued at approximately 700 XAF (USD $1.16). While participants were not informed about the gift in advance, some may have heard about it from others”.

Comment: given that some participants may have heard in advance from others about the small gift offered and certainly the questions asked, how can you be sure this did not significantly influence their responses, and introduced bias into your study? Why not share some of the questions in the text to clarify the situation?

R. We have modified the text to account for the reviewer’s comment, see Lines 373-382.

7. Results

Line 420: “vendor counts in West Dja were based on data from 16 out of the 17 surveyed markets”.

Comment: under the vendor column of Table 1, do the numbers represent number of vendors/people interviewed or the number of surveyed markets? It’s unclear.

R. The vendor counts in Table 1 represent the number of individual vendors interviewed (Lines 402-405).

8. Discussion

Lines 754 - 755: “For instance, Baka respondents were more likely than Bantu to report hunting bats”

Comment: the authors should explain briefly why the indigenous Baka were more likely than Bantu to hunt bats. Was it because bats are culturally important to the Baka which would mean some cultural behaviours engage people in some high-risk practices or was it because bats were much easier to catch?

R. Further explanation of this point has been included (Lines 702-720).

Reviewer #2:

General

1. The manuscript is an interesting study with findings that will contribute to knowledge in the wildlife value chain and zoonotic pathogen spillover.

R. We thank the reviewer for their positive assessment of the manuscript and for recognising the contribution of this study to understanding the wildlife value chain and the risk of zoonotic pathogen spillover. We appreciate the reviewer’s encouraging comments and are pleased that the findings are considered relevant to advancing knowledge in this area.

Specific

2. The authors should consider revising the title to accurately reflect the study's findings.

R. Revised title: Cultural and behavioural drivers of zoonotic disease risk along the wild meat value chain in rural Cameroon.

3. The introduction is too lengthy, and the authors should make it more concise.

R. Done. Lines 70-131.

4. The authors should specifically mention in the introduction the pathogens that spill over from wildlife to humans in Cameroon, rather than generalizing the situation in the Congo Basin.

R. We have revised the Introduction to explicitly identify zoonotic pathogens known to occur in Cameroon that are associated with wildlife and wild meat handling, rather than referring more broadly to the Congo Basin. The revised text now highlights several documented examples, including filoviruses such as Ebola and Marburg carried by fruit bats, simian retroviruses in non-human primates related to HIV, and bacterial infections such as leptospirosis, salmonellosis, and plague associated with small mammals including rodents and civets. We also clarify that human exposure can occur during activities such as hunting, butchering, transport, and food preparation, when contact with animal blood, bodily fluids, or contaminated surfaces is common. These additions make the epidemiological context of zoonotic spillover risks in Cameroon more explicit and strengthen the rationale for examining behavioural practices along wild meat value chains in the study area. We thank the reviewer for prompting this clarification, which has improved the specificity and relevance of the Introduction.

5. A limitation of this study is the classification of the wild meat value actors. A study by Saylors et al 2021 in Cameroon better classify the value chain actors as hunters, middlemen, and transporters.

R. The revised manuscript now clarifies that participants were classified according to their primary role in the wild meat value chain (hunter, vendor, or food preparer) for the purposes of the analysis. However, we recognise that in practice individuals may perform multiple roles within wildlife trade networks, and that additional actor categories—such as transporters and middlemen—can play important roles in the movement and distribution of wild meat. We now explicitly note that this classification simplifies the complexity of real-world value chains and may not fully capture the diversity of actors and interactions that shape exposure pathways and disease risk. The revised text also highlights that future research should further examine the broader range of actor types and their interactions, building on previous work (e.g., Saylors et al., 2021), to better understand how zoonotic risks emerge and propagate along wildlife trade networks.

6. The authors in lines 444-445 claim vendors consistently received wildlife from intermediaries. Please, who are these intermediaries? Be specific, are value chain actors also.

R. In this study, intermediaries refer to value chain actors who purchase wild meat directly from hunters in villages and transport or redistribute it to market vendors, typically in nearby towns or roadside markets. These individuals are often involved in the collection, aggregation, and transport of wild meat, linking rural hunters with vendors and consumers further along the trade chain. In some cases, intermediaries may also be involved in small-scale trading themselves, operating informally within local market networks. To clarify this point, we have revised the relevant section of the manuscript (Lines 244-252) to explicitly state that intermediaries are also actors within the wild meat value chain, functioning primarily as collectors or transporters who move wildlife products from hunters to vendors and other markets. This clarification helps distinguish their role from that of hunters, vendors, and household food preparers, while acknowledging their importance in the movement of wild meat within local trade networks.

7. Did the authors ask questions on the hunting method, and why was it not presented? Hunting is a major risk factor for a spillover event.

R. Information on hunting methods was indeed collected during the surveys, as these practices can influence the frequency and type of human–wildlife contact and therefore affect potential exposure to zoonotic pathogens. However, hunting methods were not included in the present analysis because the primary objective of this study was to examine risk perceptions and hygiene-related behaviours along the wild meat value chain, rather than to analyse ecological or technical aspects of hunting practices. To avoid expanding the scope of the manuscript beyond its main research questions, the analysis focused on behavioural practices directly related to handling, preparation, and consumption of wild meat. To clarify this point, we have added new text in Lines 240-242. This addition explains why these data were collected but not presented. We agree with the reviewer that hunting practices are an important factor in zoonotic spillover risk, and we plan to examine these data in future analyses focusing specifically on hunting techniques and wildlife contact patterns.

Attachment Submitted filename: Responses_Reviewers.docx

# Peer review: Decision Letter 1

**DOI:** 10.1371/journal.pone.0355275.r003

**Authors:** Pierre Roques

20 Jul 2026

Cultural and behavioural drivers of zoonotic disease risk along the wild meat value chain in rural Cameroon

PONE-D-25-28273R1

Dear Dr. Fa,

We’re pleased to inform you that your manuscript has been judged scientifically suitable for publication and will be formally accepted for publication once it meets all outstanding technical requirements.

Within one week, you’ll receive an e-mail detailing the required amendments. When these have been addressed, you’ll receive a formal acceptance letter and your manuscript will be scheduled for publication.

An invoice will be generated when your article is formally accepted. Please note, if your institution has a publishing partnership with PLOS and your article meets the relevant criteria, all or part of your publication costs will be covered. Please make sure your user information is up-to-date by logging into Editorial Manager at Editorial Manager® and clicking the ‘Update My Information' link at the top of the page. For questions related to billing, please contact billing support.

If your institution or institutions have a press office, please notify them about your upcoming paper to help maximize its impact. If they’ll be preparing press materials, please inform our press team as soon as possible -- no later than 48 hours after receiving the formal acceptance. Your manuscript will remain under strict press embargo until 2 pm Eastern Time on the date of publication. For more information, please contact onepress@plos.org.

Kind regards,

Pierre Roques, Ph.D.

Academic Editor

PLOS One

Additional Editor Comments (optional):

Reviewers' comments:

Reviewer's Responses to Questions

**Comments to the Author**

1. If the authors have adequately addressed your comments raised in a previous round of review and you feel that this manuscript is now acceptable for publication, you may indicate that here to bypass the “Comments to the Author” section, enter your conflict of interest statement in the “Confidential to Editor” section, and submit your "Accept" recommendation.

Reviewer #1: All comments have been addressed

Reviewer #2: All comments have been addressed

**********

2. Is the manuscript technically sound, and do the data support the conclusions?

The manuscript must describe a technically sound piece of scientific research with data that supports the conclusions. Experiments must have been conducted rigorously, with appropriate controls, replication, and sample sizes. The conclusions must be drawn appropriately based on the data presented.

Reviewer #1: Yes

Reviewer #2: Yes

**********

3. Has the statistical analysis been performed appropriately and rigorously?

Reviewer #1: Yes

Reviewer #2: Yes

**********

4. Have the authors made all data underlying the findings in their manuscript fully available?

The PLOS Data policy requires authors to make all data underlying the findings described in their manuscript fully available without restriction, with rare exception (please refer to the Data Availability Statement in the manuscript PDF file). The data should be provided as part of the manuscript or its supporting information, or deposited to a public repository. For example, in addition to summary statistics, the data points behind means, medians and variance measures should be available. If there are restrictions on publicly sharing data—e.g. participant privacy or use of data from a third party—those must be specified.

Reviewer #1: Yes

Reviewer #2: Yes

**********

5. Is the manuscript presented in an intelligible fashion and written in standard English?

PLOS ONE does not copyedit accepted manuscripts, so the language in submitted articles must be clear, correct, and unambiguous. Any typographical or grammatical errors should be corrected at revision, so please note any specific errors here.

Reviewer #1: Yes

Reviewer #2: Yes

**********

6. Review Comments to the Author

Please use the space provided to explain your answers to the questions above. You may also include additional comments for the author, including concerns about dual publication, research ethics, or publication ethics. (Please upload your review as an attachment if it exceeds 20,000 characters)

Reviewer #1: (No Response)

Reviewer #2: All comments have been adequately addressed by the authors. Therefore, I have no further comments for the authors

**********

7. PLOS authors have the option to publish the peer review history of their article (what does this mean?). If published, this will include your full peer review and any attached files.

If you choose “no”, your identity will remain anonymous but your review may still be made public.

**Do you want your identity to be public for this peer review?**  For information about this choice, including consent withdrawal, please see our Privacy Policy.

Reviewer #1: **Yes:** Eric Djomo Nana

Reviewer #2: **Yes:** Adeyinka Jeremy Adedeji

**********

# Editor report: Acceptance letter

**DOI:** 10.1371/journal.pone.0355275.r004

**Authors:** Pierre Roques

PONE-D-25-28273R1

PLOS One

Dear Dr. Fa,

I'm pleased to inform you that your manuscript has been deemed suitable for publication in PLOS One. Congratulations! Your manuscript is now being handed over to our production team.

At this stage, our production department will prepare your paper for publication. This includes ensuring the following:

* All references, tables, and figures are properly cited

* All relevant supporting information is included in the manuscript submission,

* There are no issues that prevent the paper from being properly typeset

You will receive further instructions from the production team, including instructions on how to review your proof when it is ready. Please keep in mind that we are working through a large volume of accepted articles, so please give us a few days to review your paper and let you know the next and final steps.

Lastly, if your institution or institutions have a press office, please let them know about your upcoming paper now to help maximize its impact. If they'll be preparing press materials, please inform our press team within the next 48 hours. Your manuscript will remain under strict press embargo until 2 pm Eastern Time on the date of publication. For more information, please contact onepress@plos.org.

You will receive an invoice from PLOS for your publication fee after your manuscript has reached the completed accept phase. If you receive an email requesting payment before acceptance or for any other service, this may be a phishing scheme. Learn how to identify phishing emails and protect your accounts at https://explore.plos.org/phishing.

If we can help with anything else, please email us at customercare@plos.org.

Thank you for submitting your work to PLOS ONE and supporting open access.

Kind regards,

PLOS ONE Editorial Office Staff

on behalf of

Dr. Pierre Roques

Academic Editor

PLOS One
